# Parity and gestational age are associated with vaginal microbiota composition in term and late term pregnancies

**DOI:** 10.1016/j.ebiom.2022.104107

**Published:** 2022-06-24

**Authors:** Kaisa Kervinen, Tiina Holster, Schahzad Saqib, Seppo Virtanen, Vedran Stefanovic, Leena Rahkonen, Pekka Nieminen, Anne Salonen, Ilkka Kalliala

**Affiliations:** a Department of Obstetrics and Gynaecology, University of Helsinki and Helsinki University Hospital, Helsinki, Finland; b Human Microbiome Research Program, Faculty of Medicine, University of Helsinki, Helsinki, Finland; c Department of Metabolism, Digestion and Reproduction, Faculty of Medicine, Imperial College London, London, UK

**Keywords:** Microbiota, Pregnancy, Nulliparous, Vagina, Gestational age

## Abstract

**Background:**

Vaginal microbiota and its potential contribution to preterm birth is under intense research. However, only few studies have investigated the vaginal microbiota in later stages of pregnancy or at the onset of labour.

**Methods:**

We used 16S rRNA gene amplicon sequencing to analyse cross-sectional vaginal swab samples from 324 Finnish women between 37–42 weeks of gestation, sampled before elective caesarean section, at the onset of spontaneous labour, and in pregnancies lasting ≥41 weeks of gestation. Microbiota data were combined with comprehensive clinical data to identify factors associated with microbiota variation.

**Findings:**

Vaginal microbiota composition associated strongly with advancing gestational age and parity, i.e. presence of previous deliveries. Absence of previous deliveries was a strong predictor of *Lactobacillus crispatus* dominated vaginal microbiota, and the relative abundance of *L. crispatus* was higher in late term pregnancies, especially among nulliparous women.

**Interpretation:**

This study identified late term pregnancy and reproductive history as factors underlying high abundance of gynaecological health-associated *L. crispatus* in pregnant women. Our results suggest that the vaginal microbiota affects or reflects the regulation of the duration of gestation and labour onset, with potentially vast clinical utilities. Further studies are needed to address the causality and the mechanisms on how previous labour, but not pregnancy, affects the vaginal microbiota. Parity and gestational age should be accounted for in future studies on vaginal microbiota and reproductive outcomes.

**Funding:**

This research was supported by EU H2020 programme Sweet Crosstalk ITN (814102), Academy of Finland, State Research Funding, and University of Helsinki.

**Research in context**

**Evidence before this study**

Certain bacteria, such as *Lactobacillus iners,* have been associated with preterm birth, whereas *L. crispatus* has been seen to favour term delivery. However, as most studies have focused on vaginal microbiota in the first or second trimester of pregnancy, there is lack of knowledge on the characteristics of the vaginal microbiota in later stages of pregnancy, and whether the composition of microbiota differs in term and late term pregnancies. There is also emerging evidence for the potential role of pregnancy history among the factors associated with the vaginal microbiota in the first trimester of subsequent pregnancies, and that it may have an impact on the vaginal microbiota of reproductive aged women in general.

**Added value of this study**

Studies assessing the vaginal microbiota composition specifically in term (37–40 gestational weeks) and late term (≥41 gestational weeks) pregnancy are rare. We show that pregnancy history has an influence on the vaginal microbiota at the late stages of pregnancy, and that rising gestational age is associated with higher relative abundances of *L. crispatus*, especially in women without previous deliveries.

**Implications of all the available evidence**

Our results suggest that there is a possible connection between vaginal microbiota composition and prolonged pregnancy. Our results also support previous findings showing that pregnancy history is an important factor affecting the vaginal microbiota composition during different stages of the subsequent pregnancy and this should be regarded in future studies on vaginal microbiota.

Alt-text: Unlabelled box

## Introduction

The vaginal microbial environment is affected and modified by multiple factors.^1^, ^2^, ^3^, ^4^, ^5^ During pregnancy vaginal microbiota is often dominated by the genus *Lactobacillus*,^6^ and the microbiota is more stable and less diverse compared to the non‐pregnant state.^7^^,^^8^ Many factors have been hypothesized to explain this phenomenon, including the lack of cyclic hormonal fluctuations and menstruation, changes in cervicovaginal secretions, and decreased sexual activity.^7^ Rising oestrogen levels during pregnancy result in increasing vaginal glycogen accumulation, favouring the proliferation of lactobacilli^9^ which protect the upper genital tract from ascending pathogens.

While the drastic changes in the vaginal microbiota on transition from pregnancy to post-partum state are well described,^10^ only couple of studies have addressed the potential lasting effects of pregnancy and childbirth on women's vaginal microbiota composition. A US-based study showed that number of previous pregnancies was associated with vaginal microbiota composition in the first trimester of pregnancy,^11^ while a recent large study in healthy Chinese non-pregnant, reproductive age women reported that pregnancy history is essentially the strongest influencer of the vaginal microbiota, surpassing the effect of e.g. menstrual cycle.^5^ In both studies, dominance of *Lactobacillus crispatus* was found to be highest in women without previous conception.

Average human gestation lasts 40 weeks (280 days). Term pregnancy can be defined as early term (37 0/7 through 38 6/7 weeks of gestation (GW), full term (GW 39 0/7 through 40 6/7), late term (GW 41 0/7 through 41 6/7), and post term (GW 42 0/7 and beyond).^12^ The vaginal microbiota differs between women giving birth prematurely or at term.^13^ Studies on predominantly Caucasian populations have reported that increased diversity, dominance by *Lactobacillus iners* or overall depletion of *Lactobacillus* spp. increase the risk for preterm birth (PTB),^14^, ^15^, ^16^, ^17^ whereas dominance of *Lactobacillus crispatus* in the vaginal microbiota would act as protection against PTB.^15^^,^^18^ Vaginal microbiota has been suggested to change with increasing gestational age with the relative abundance of *Lactobacillus* spp. possibly declining towards child birth,^19^^,^^20^ and in late gestation to have similarities to the non-pregnant microbiota.^6^

The mechanisms behind the onset of spontaneous labour, as well as factors determining the duration of gestation remain largely unknown.^21^ Late term and post term pregnancy, i.e. gestation lasting more than 41 weeks is associated with maternal and foetal risks, higher rates of operative delivery, and increased perinatal mortality.^22^ In Western countries, induction of labour is performed in almost 50% of all pregnancies that proceed beyond 41 weeks,^23^ of which roughly 20% end up in caesarean section (CS)^24^ with the risk being higher among nulliparous women, i.e. women with no previous deliveries.^25^^,^^26^ However, studies investigating microbial composition with increasing duration of pregnancy are scarce.^20^

Our objective was to assess the vaginal microbiota in full and late term pregnancy with an aim to study whether gestational age or other host-related factors influence the composition of vaginal microbiota at or near delivery, and whether vaginal microbiota differs according to previous pregnancy outcomes.

## Methods

### Ethics and recruitment of study subjects

The study was carried out at the Department of Obstetrics and Gynaecology, Helsinki University Hospital, Helsinki, Finland. Patients were recruited and the samples were collected between May 2017 and December 2018. The study was approved by the ethical committee of the Hospital District of Helsinki and Uusimaa (HUS/907/2017) and performed in accordance with the principles of the Helsinki Declaration. All participants signed an informed consent and participation was voluntary.

**Figure 1:**
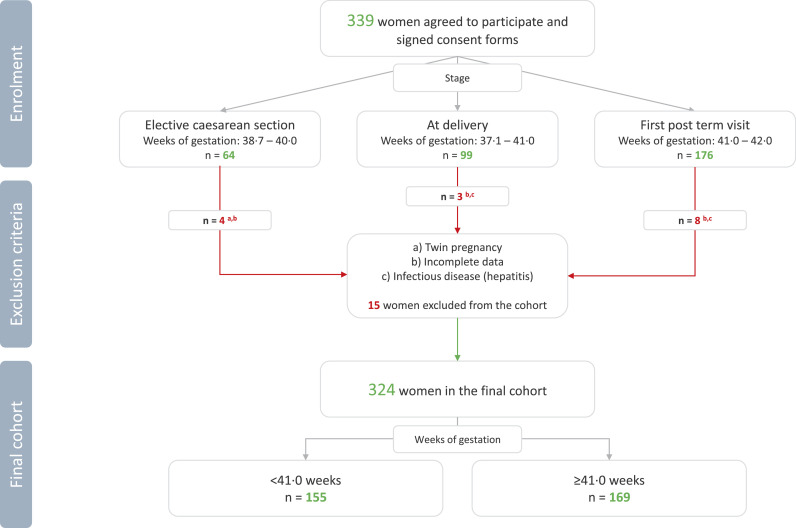
Flowchart of the study population. Altogether 339 women were recruited to take part in the study at the time of planned elective caesarean section, on admittance to the delivery ward due to contractions, and at the time of appointment when pregnancy proceeded beyond due date. After excluding women based on set exclusion criteria the final study cohort consisted of 324 women.

Women aged 20 to 47 years were recruited at the time of planned elective CS, on admittance to the delivery ward due to contractions (i.e. women at the first stage of labour), or at the time of appointment when pregnancy proceeded beyond due date. According to the current departmental management guidelines, all women with an uncomplicated pregnancy receive an appointment for an antenatal visit in the maternity outpatient clinic latest at 41 5/7 weeks of gestation. A flow chart for selection of study population is shown in Figure 1. Exclusion criteria included age under 18 years, multifetal gestation, maternal type 1 and type 2 diabetes, placenta-related pregnancy complications (e.g. pre-eclampsia and/or intrauterine growth restriction), and maternal bloodborne infectious diseases (viral hepatitis, HIV). Women with incomplete medical record data (missing at random – MAR) were also excluded. The final study population included 324 women.

The participants were asked to complete a background questionnaire requesting information about their gynaecological history, sexual habits, previous infections, antibiotic and probiotic use, relationship status, and educational status. Maternal pre-pregnancy body mass index (BMI), pregnancy history, smoking habits (current and/or previous smoking), postpartum infections and gestational age at sampling were obtained from antenatal clinic charts. A pregnancy with gestational age at sampling of under 41 weeks of gestation was considered term and of 41 weeks and beyond late term. Gestational age was determined by the crown-rump-length measurement at the time of the first trimester ultrasound screening. Women were categorised as multiparous in the presence of any number of previous deliveries in their clinical history and nulliparous if there were none. Nulliparous women were further sub-categorised as primigravida if they had never been pregnant and nulliparous multigravida if they had a history of spontaneous/induced abortion(s). Degree of education was reported as five-class: comprehensive school, vocational upper secondary education, technical college, general upper secondary education, and tertiary degree from university or university of applied sciences. A tertiary degree was defined as higher education. The number of sex partners during lifetime was reported as dichotomous (more than three partners or less) and ordinal (1-3, 4-10, 11-20, >20). Postpartum infections were defined as infections requiring antibiotic treatment within two weeks after delivery and comprised of endometritis, episiotomy infections, post-caesarean wound infections, and urinary tract infections. Time since previous pregnancy was counted in months from the sampling date to the end date of the previous pregnancy, which was either the date of previous delivery or the date when last abortion was diagnosed/induced.

Mid vaginal wall samples were collected with sterile flocked swabs (FLOQSwabs, CP520CS01, Copan Flock Technologies, Italy) by healthcare professionals at the time of planned elective caesarean section, on admittance to the delivery ward due to contractions (i.e. women at the first stage of labour), or at the time of appointment when pregnancy proceeded beyond due date. All women had intact foetal membranes at the time of sampling. Tips of the swabs were severed to 1·5 mL Eppendorf tubes without medium and were frozen in −20 °C immediately after sampling. The samples were further moved to −80 °C within two weeks. The median time the samples were in -80 °C before sequencing was 10·9 months (Interquartile range, IQR 6·7–13·3).

### DNA extraction and sequencing of the 16S rRNA gene amplicons

Bacterial DNA was extracted from the swabs using a bead beating method, profiled for quality, used as template for amplification of the V3-V4 region of the 16S rRNA gene (using primers 341F 5′-CCTACGGGNGGCWGCAG-3′ and 785Rev 5′-GACTACHVGGGTATCTAATCC-3′), and subsequent construction of indexed libraries compatible for multiplexed Illumina MiSeq sequencing as previously described.^4^ Samples were sequenced in four different runs, the samples from different study groups being randomly assigned to the runs. Part of the samples were sequenced together with fungal amplicons; in that case the bacterial reads were first extracted from the fastq-files with cutadapt (python 3.8.5) based on primer sequences.^27^ No run ID based batch effects on the microbiota were observed in permutational ANOVA (F=1·15, R2= 0·010, *p*=0·30).

### Sequence pre-processing and analysis

The paired-end sequencing data was pre-processed using the dada2 R package (1.20)^28^ and its accompanying workflow (tutorial 1.16). The amplicon sequence variants (ASVs) obtained from dada2 were annotated using the taxminer R package workflow.^29^ This consists of BLAST based sequence alignments followed by a text-mining based strategy that attaches ecosystem specificity to each alignment (host & isolation source) to probabilistically select the most likely taxonomic annotations.

### Statistical analysis

Statistical analyses were performed using R (version 4.1.2).^30^ Permutational multivariate analysis of variance (PERMANOVA; adonis function in the vegan package^31^) based on the Bray-Curtis distance was used to identify factors that explain between-sample variation in the microbiota (beta-diversity, Table S1). Comparison of categorical variables was measured with two-tailed Chi square and Fisher's exact tests when appropriate, and continuous variables with two-tailed t-test when normally distributed and two-tailed Mann Whitney U-test for non-normally distributed variables. GroupTest and CovariateTest from the mare R package^32^ were used for species-wise comparisons between subgroups within the cohort, and for studying associations between clinical variables and individual taxa, respectively. These functions sequentially apply different statistical models along with appropriate transformations (relative abundance, log transformation), selecting the optimal model for each taxon based on the data distributions and model performance/fit. The models selected and used within this analysis include linear model (lm) and generalized linear model (glm) from the stats package,^30^ negative binomial generalized linear model (glm.nb) from the MASS package^33^ and linear model using generalized least squares (gls) from the nlme package.^34^ Based on the quality of model fit, if none of the models being applied were found to be appropriate for the data, no *p*-value is reported. A minimum acceptable prevalence and relative abundance of a taxon was set to 5%. The statistical models of mare functions use sample read count as an offset and *p*-values are corrected for false discovery rate (FDR; Benjamini-Hochberg).^35^ FDR-corrected *p*-values are reported in the text as *q*-values, and a value <0·05 was considered statistically significant. Biologically relevant background variables such as probiotic use and BMI were tested as potential confounders using PERMANOVA analysis, while directed acyclic graphs (DAG) were produced to identify the presence of confounding variables and potential effect modifiers within the clinical and background variables.^36^ According to DAGs, age was identified as a confounder regarding the association between parity and vaginal microbiota, and the variables age, BMI, smoking and nulliparity regarding the association between gestational age and vaginal microbiota. All figures were created in RStudio using ggplot2,^37^ gghalves,^38^ cowplot^39^ and metacoder^40^ R packages.

### Sample size estimation

Power calculations were not applicable to our study setting since earlier studies on comparing the vaginal microbiota in term versus late term pregnancies are lacking.

### Role of the funding source

The funders were not involved in study design, data collection, analysis, interpretation, or writing.

## Results

### Cohort characteristics

A total of 324 pregnant women were included in the study, of which 155 (47·8%) were under 41.0 weeks of gestation and 169 (52·2%) were at or exceeded 41.0 weeks of gestation. Of the samples, 60 (18·5%) were taken at elective CS (GW between 38·7–40·4), 96 at delivery (29·6%, GW between 37·1–41·0) and 168 (51·8%) at post term visit at maternal outpatient clinic (GW between 41·0–42·0). The demographic, lifestyle and clinical characteristics of the study population are shown in Table 1 and in Supplementary Table 1. Of all studied women, 58·0% (n=188) were nulliparous and of these 139 (73·9%) had no previous pregnancies and 49 (26·1%) had at least one miscarriage or medical abortion in their pregnancy history. The median gestational age at sampling was 41·0 weeks (IQR 39·7–41·7), the mean age 32·1 years (SD 4·8), and the median BMI 23·1 kg/m^2^ (IQR 21·2–26·4).

**Table 1:** Characteristics of the study population.

		Parity			Gestational age (weeks)		
Characteristics	All	Nulliparous	Multiparous	*p*-value	<41·0	≥41·0	*p*-value
Number of women	324	188	136		155	169	
Age, years							
Mean (SD, range)^1^	32·1 (4·8, 20–47)	31·6 (4·7, 20–43)	32·8 (4·8, 21–47)	**0·016**	31·9 (4·7, 20–47)	32·2 (4·9, 20–43)	0·51
BMI, kg/m^2^							
Median (IQR)^2^	23·1 (21·2–26·4)	23·1 (21·3–26·5)	23·1 (21·2–26·0)	0·91	23·0 (21·0–25·0)	24·0 (21·5–27·3)	**0·022**
Nullipara^3^	188/324 (58·0)				82/155 (52·9)	106/169 (62·7)	0·074
Primigravida	139/188 (73·9)				60/82 (73·2)	79/106 (74·5)	0·83
Nulliparous multigravida	49/188 (26·1)				22/82 (26·8)	27/106 (25·5)	
Current smoking^3^	10/324 (3·1)	2/188 (1·1)	8/136 (5·9)	**0·013**	7/155 (4·5)	3/169 (1·8)	0·20
Infertility treatments (current pregnancy)^3^	22/324 (6·8)	17/188 (9·0)	5/136 (3·7)	0·058	16/155 (10·3)	6/169 (3·6)	**0·015**
Use of probiotics*^,^^3^	192/303 (63·4)	107/171 (62·6)	85/132 (64·4)	0·74	88/141 (62·4)	104/162 (64·2)	0·75
Antibiotic use (<3 months)*^,^^3^	45/302 (14·9)	23/172 (13·4)	22/130 (16·9)	0·39	16/140 (11·4)	29/162 (17·9)	0·12
Intercourse <48h*^,^^3^	46/304 (15·1)	26/174 (14·9)	20/130 (15·4)	0·92	15/142 (10·6)	31/162 (19·1)	**0·037**
High education (tertiary degree)*^,^^3^	198/303 (65·3)	117/171 (68·4)	81/132 (61·4)	0·20	107/164 (65·2)	91/139 (65·5)	0·97
Married or cohabiting*^,^^3^	288/304 (94·7)	164/172 (95·3)	124/132 (93·9)	0·59	133/139 (95·7)	155/165 (93·9)	0·50
Pregnancy related characteristics							
Gestational age at sampling, weeks							
Median (IQR)^2^	41·0 (39·7–41·7)	41·4 (40·1–41·7)	40·6 (39·4–41·7)	**0·027**	39·7 (39·1–40·3)	41·7 (41·6–41·7)	**<0·0001**
≥H41+0^3^	169/324 (52·2)	106/188 (56·4)	63/136 (46·3)	0·074			
Gestational age at birth, weeks							
Median (IQR)^2^	41·2 (39·7–41·9)	41·6 (40·1–42·0)	40·6 (39·4–41·9)	**0·0014**	39·7 (39·1–40·4)	41·9 (41·9–42·0)	**<0·0001**
Contractions at sampling^3^	103/324 (31·8)	63/188 (33·5)	40/136 (29·4)	0·43	95/155 (61·3)	8/169 (4·7)	**<0·0001**
Gestational diabetes^3^	79/324 (24·4)	46/188 (24·5)	33/136 (24·3)	0·97	37/155 (23·9)	42/169 (24·9)	0·84
Maternal postpartum infection†^,^^3^	32/324 (9·9)	18/188 (9·6)	14/136 (10·3)	0·83	11/155 (7·1)	21/169 (12·4)	0·11
Birthweight, g							
Mean (SD, range)^1^	3679 (474, 2636–5130)	3627 (445, 2690–5020)	3751 (502, 2636–5130)	**0·020**	3538 (421, 2636–4744)	3808 (2740–5130)	**<0·0001**

BMI, body mass index; SD, standard deviation; IQR, interquartile range.

Data are n (%) unless otherwise specified.

⁎ Missing information for data collected from questionnaires, total number of women for which data is known is shown for each variable.

† Postpartum infections included endometritis n=24, episiotomy infection n=6, urinary tract infection n=1, post-caesarean section wound infection n=1.

1 t test.

2 Mann-Whitney U-test.

3 Chi square and Fisher's exact test when appropriate.

### Vaginal microbiota in late pregnancy in Finnish women

From this point forth, “presence/detection” of a bacterium is defined as >5% and “dominance” is defined as >50% of relative abundance in the sample. Lactobacilli were detected in 309/324 (95·4%) women (Supplementary Table 2), of which 256/324 (79·0%) had *Lactobacillus*-dominated vaginal microbiota (Supplementary Table 3). *L. crispatus* was the most abundant and prevalent species overall, being present in 170/324 (52·5%) of the samples (Supplementary Table 4) and the dominant bacterium of the vaginal microbiota of 142/324 (43·8%) of women (Supplementary Table 5). *L. iners* was detected in 119/324 (36·7%) and was the dominant species in 90/324 (27·8%) women. Other *Lactobacillus* species of note were *L. jensenii* (detected: 48/324, 14·8%; dominant: 12/324, 3·7%; Supplementary Tables 4–5) and *L. gasseri* (detected: 31/324; 9·6%, dominant: 12/324, 3·7%; Supplementary Tables 4–5). *Gardnerella* spp. were the most prevalent non-*Lactobacillus* bacteria and were present in 81/324 (25·0%) and dominant in 30/324 (9·3%) women (Supplementary Tables 4–5). Two species of *Gardnerella* were detected, *G. vaginalis* (detected: 78/324, 24·1%; dominant: 29/324, 9·0%; Supplementary Tables 4–5) and *G. leopoldii* (detected: 18/324, 5·6%; dominant: 1/324, 0·3%; Supplementary Tables 4–5). Altogether 55 different species-level taxa were detected, representing 24 genera and 7 higher taxonomic ranks (Supplementary Fig. 1).

### Vaginal microbiota and parity

**Figure 2:**
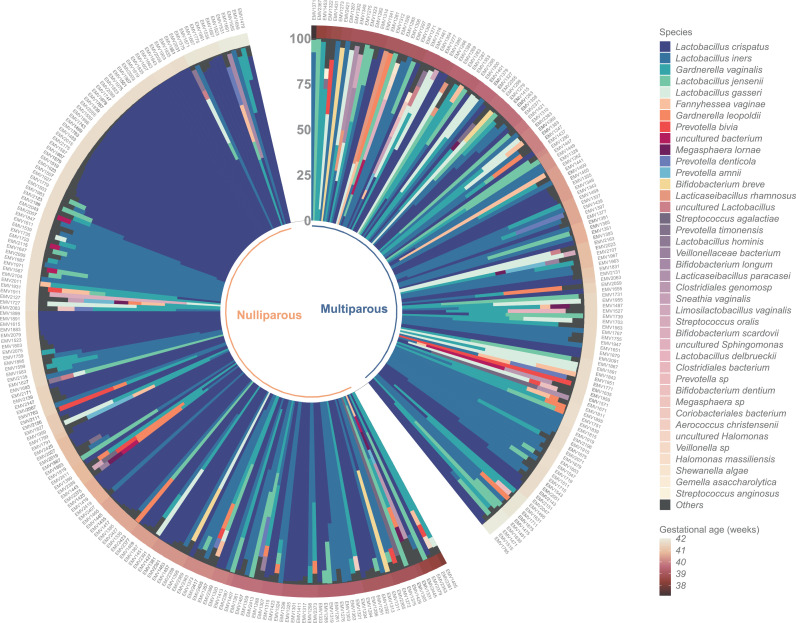
Relative abundances of bacterial taxa across all 324 samples. Circular stacked bar chart showing the bacterial relative abundances of all the samples in the study cohort (n=324), divided based on parity – no previous pregnancies or deliveries (nulliparous) (n=188) and one or more previous deliveries (multiparous) (n=136). The samples have been ordered based on two criteria 1) gestational age in weeks, increasing clockwise (outer band) and 2) within each gestational age bracket, whether one of the top 3 bacteria were the most abundant (>50% composition), ordered clockwise from None, to *Gardnerella vaginalis, Lactobacillus iners*, and *Lactobacillus crispatus* dominant samples.

**Figure 3:**
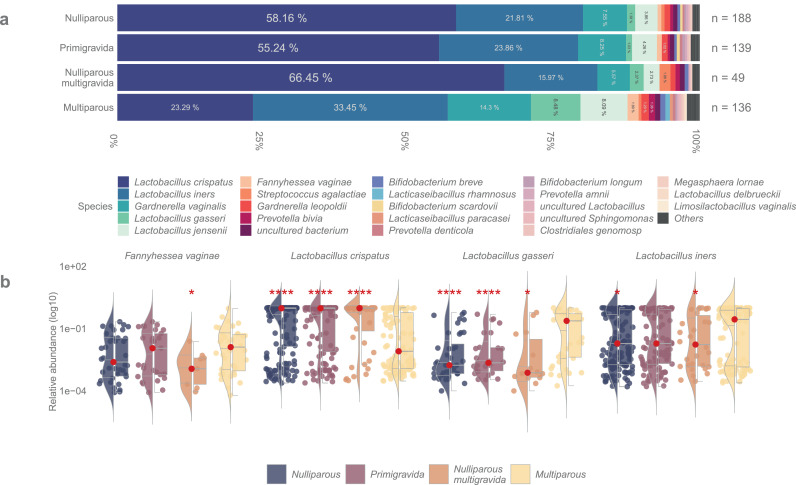
Abundance of bacterial taxa in pregnant women based on previous pregnancy history. (a) Stacked bar plot depicting the mean bacterial relative abundances of samples from women without prior pregnancies or deliveries (nulliparous) (n=188), women without prior pregnancies (primigravida) (n=139), women with prior spontaneous or induced abortion (nulliparous multigravida) (n=49), and women with previous deliveries (multiparous) (n=136). (b) Violin + box + jitter plots showing the distribution of taxa (log10 relative abundance) with significantly different abundances between the sample groups. The whiskers on the boxplot represent the 1·5 interquartile range and the median value is shown as a red dot. The horizontal lines on the violin plots represent the 25^th^, 50^th^, and 75^th^ quantiles. Each point on the jitter plot represents a sample, highlighting the density and frequency of occurrence of a taxon. Asterisks indicate whether there were statistically significant differences between the subgroups compared to the multiparous samples, *q* <0·0001, *q* <0·001, *q* <0·01, *q* <0·05. The following models were used within this analysis: glm·nb for *Fannyhessea vaginae* and *Lactobacillus gasseri*; log gls for *Lactobacillus crispatus* and *Lactobacillus iners*.

The gross vaginal microbiota composition associated strongly with parity (PERMANOVA F=21·30; R2=0·062; p<0·0001, Supplementary Table 6). The bacterial profiles of samples stratified by parity are shown in Figure 2. *L. crispatus* was more frequent in nulliparas (detected: 123/188, 65·4%; dominant: 111/188, 59·0%) than in multiparas (detected: 47/136, 34·6%; dominant: 31/136, 22·8%) (Figure 2; Supplementary Tables 4–5). The mean relative abundance of *L. crispatus* was 2·5 times more abundant in samples from nulliparous women compared to multipara (58·2% vs. 23·3%, q<0·0001, gls, Figure 3, Supplementary Table 7). On the contrary, *L. iners, L. gasseri, G. vaginalis,* and *Fannyhessea vaginae* (previously *Atopobium vaginae*) were more commonly seen in multiparas (33·5% in multiparas vs. 21·8% in nulliparas, *q*=0·029, gls; 8·48% vs. 1·38%, *q*<0·0001, glm.nb; 14·3% vs. 7·55%, q>0·05, gls; 1·89% vs. 0·64%, *q*>0·05, glm.nb, respectively; Supplementary Table 7). The results remained after adjusting for age (Supplementary Table 8).

To study the effect of previous pregnancies vs. deliveries, the nulliparous women were further stratified into primigravidas (no previous pregnancies) and nulliparous multigravidas (presence of previous pregnancies that ended in spontaneous or induced abortion). No significant differences in relative abundances of any bacteria were observed between these two groups (Figure 3, Supplementary Fig. 2, Supplementary Table 7). *L. crispatus* was the most abundant bacterium overall but was detected more often in nulliparous multigravidas (mean relative abundance nulliparous multigravidas: 66·5% vs. primigravidas: 55·2%, *q*=0·19, gls). The distribution of *L. crispatus* in both stratified subgroups of nulliparas compared to multiparas was consistently significant (*q*<0·0001, gls) as was that of *L. gasseri* (mean relative abundance primigravidas: 1·03%, *q*<0·0001, glm.nb; nulliparous multigravidas: 2·37%, *q*=0·19, glm.nb). The mean relative abundance of *L. iners* in nulliparous multigravidas (16·0%) was lower than in the primigravidas’ samples (23·9%, *q*=0·19, gls) and significantly lower than among multiparas (*q*=0·010, gls). All these results remained after adjusting for age (Supplementary Table 8). Only trace amounts of *F. vaginae* (<1%) were observed in nulliparous multigravidas’ samples (*q*=0·010, glm.nb).

In women with one prior delivery compared to nulliparas, the differences in the relative abundances of *L. crispatus* and *L. gasseri* were only observed after vaginal delivery or birth by emergency CS (*q*<0·001, gls; *q*<0·001, glm.nb, respectively), whereas no significant differences in their abundances were seen between nulliparas and women with one previous delivery by elective CS (Supplementary Fig. 3, Supplementary Table 7). However, the sample size of women with one previous delivery by elective CS was small (n=8).

The number of previous deliveries correlated negatively with abundance of *L. crispatus*: the mean relative abundance decreased from 58·1% in nulliparas to 25·7% in women with one prior delivery and to 15·4% with two or more deliveries (*q*<0·0001, gls, Supplementary Fig. 4, Supplementary Table 7). On the other hand, the relative abundances of *L. gasseri* and *L. iners* increased with rising number of previous deliveries (*q*<0·0001, glm.nb and *q*=0·046, gls, respectively, Supplementary Table 7). The abundance of *G. vaginalis* was seen to decrease (*q*=0·039, gls) with increasing intervals between previous delivery and current pregnancy, but no differences in *Lactobacillus* abundances were observed in relation to time since last delivery (Supplementary Table 7).

### Vaginal microbiota and increasing gestational weeks

**Figure 4:**
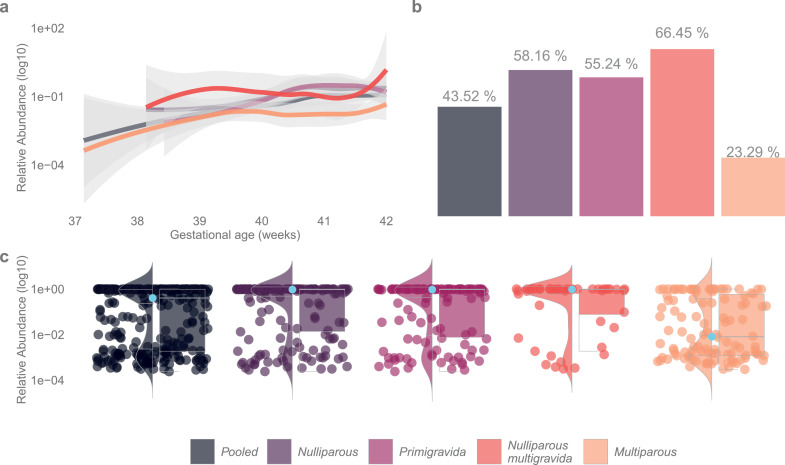
*Lactobacillus crispatus* distribution in relation to gestational age and parity. Summary of *L. crispatus* distribution across all samples (pooled, n=324), women with no previous pregnancies or deliveries (nulliparous) (n=188), women without prior pregnancies (primigravida) (n=139), women with prior spontaneous or induced abortion (nulliparous multigravida) (n=49), and women with one or more previous deliveries (multiparous) (n=136). (a) Local regression models representing the change in relative abundance (log10 scale) of *L. crispatus* with increasing gestational age in weeks. (b) Bar plot showing the mean relative abundance of *L. crispatus* in each subgroup across all gestational weeks. (c) Violin + box + jitter plots showing the distribution of taxa (log10 relative abundance) with significantly different abundances between the sample groups. The whiskers on the boxplot represent the 1·5 interquartile range and the median value is shown as a sky-blue dot. The horizontal lines on the violin plots represent the 25th, 50th, and 75th quantiles. Each point/dash on the jitter plot represents a sample, highlighting the density and frequency of occurrence of *L. crispatus*.

Gestational age at the time of sampling associated significantly with microbiota variation (PERMANOVA F=4·44, R2=0·014, *p*=0·0067, Supplementary Table 6). *L. crispatus* was the most abundant bacterium in both term (GW <41·0) and late term (GW ≥41·0) samples (mean relative abundance 37·5% and 49·0%, respectively, *q*=0·33, gls), but a significant increase in the relative abundance of *L. crispatus* was seen with advancing gestational age (*q*=0·036, gls, Figure 4, Supplementary Table 9).

When stratified according to parity, the rise in the abundance of *L. crispatus* with increasing gestational age was only evident in nulliparas (*q*=0·045, gls), not in multiparas (Figure 4, Supplementary Table 9), and more precisely among primigravidas (*q*=0·016, gls, Supplementary Table 9). Among primigravidas the relative abundance of *L. gasseri* increased (*q*<0·0001, glm.nb) while the abundance of *L. iners* and *L. jensenii* declined along gestational age (*q*=0·030, gls and *q*=0·016, glm.nb, respectively), whereas for nulliparous multigravidas the amount of *L. gasseri* significantly decreased (q<0·0001, glm.nb). No differences in bacterial relative abundances were observed among multiparas with rising gestational age (Supplementary Table 9). The results remained the same after adjusting for age BMI and smoking (Supplementary Table 10).

**Figure 5:**
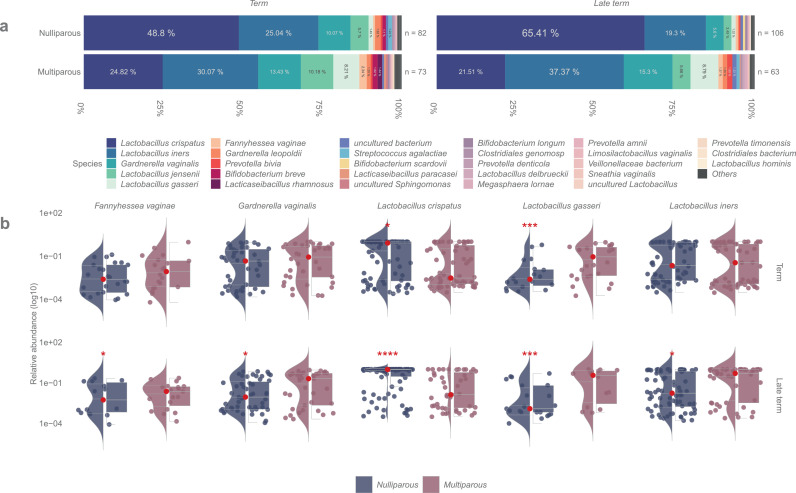
Relative abundances of bacterial taxa based on gestational age and parity. (a) Stacked bar plot depicting the mean bacterial relative abundances of samples from women whose duration of pregnancy was <41·0 gestation weeks (Term) (n=155) or >41 weeks (Late term) (n=169). The graphic is further split based on parity - no previous deliveries (nulliparous) (n=188) and one or more previous deliveries (multiparous) (n=136). (b) Violin + box + jitter plots showing the distribution of taxa (log10 relative abundance) with significantly different abundances between nulli- and multiparous in term and late term groups. The whiskers on the boxplot represent the 1·5 interquartile range and the median value is shown as a red dot. The horizontal lines on the violin plots represent the 25^th^, 50^th^, and 75^th^ quantiles. Each point on the jitter plot represents a sample, highlighting the density and frequency of occurrence of a taxon. Asterisks indicate whether there were statistically significant differences between the subgroups compared to the multiparous samples, *q* <0·0001, *q* <0·001, *q* <0·01, *q* <0·05. The following models were used in this analysis: 1) Term – glm·nb for *Fannyhessea vaginae* and *Lactobacillus gasseri*; log gls for *Gardnerella vaginalis, Lactobacillus crispatus*, and *Lactobacillus iners* 2) Late term - glm·nb for *Lactobacillus gasseri*; gls for *Lactobacillus crispatus*; log glsfor *Fannyhessea vaginae, Gardnerella vaginalis, Lactobacillus iners*.

The parity related differences in the vaginal microbiota were more distinct in late term samples compared to samples taken at term with significant differences in *L. crispatus* (*q*<0·0001, gls), *L. gasseri* (*q*<0·001, glm.nb), *L. iners* (*q*=0·037, gls), *G. vaginalis* (*q*=0·037, gls) and *F. vaginae* (*q*=0·025, gls) relative abundances between nulliparas and multiparas (Figure 5, Supplementary Table 9).

### Associations between microbiological and demographic characteristics

The associations of demographic variables with microbiota variation and single bacterial taxa are shown in Supplementary Table 11. Education level correlated significantly with the overall microbiota variation (PERMANOVA F=2·50, R2=0·032, *p*=0·0037, Supplementary Table 6) and higher education was associated with lower relative abundance of *L. iners* (*q*<0·001, gls), *G. vaginalis* (*q*=0·0054, gls) and *G. leopoldii* (*q*<0·0001, glm.nb). Smoking was associated with higher abundance of *L. iners* (*q*=0·0039, gls) as was higher amount of lifetime sex partners (identical observations in both dichotomous and ordinal variables, dichotomous reported for simplicity: *q*=0·0012, gls), whereas higher BMI was associated with higher abundance of *G. vaginalis* (*q*=0·017, gls). Smoking was also related to lower abundance of *L. gasseri* (*q*<0·001, glm.nb). History of fertility treatments either related to current pregnancy or in the past was associated with lower prevalence of *F. vaginae* (*q*<0·0001, glm.nb). Intercourse <48 hours prior to sampling associated with lower abundance of *L. gasseri* (*q*=0·013, glm.nb). Use of probiotics associated with lower levels of *G. leopoldii* (*q*=0·0026, glm.nb). The groups in which the samples were collected (elective CS, during delivery, first post term antenatal visit) did not correlate with gross microbiota variation (PERMANOVA F=1·23, R2=0·008, *p*=0·27, Supplementary Table 6) and no differences in abundances of bacteria between groups was seen. Maternal age, previous preterm births, gestational diabetes in current pregnancy, reported gynaecological infections, use of antibiotics in the recent three months, postpartum infections, or contractions at the time of sample did not associate significantly with the overall microbiota variation (Supplementary Table 6) or abundance of any individual bacterium.

## Discussion

The characteristics and individual variations of the bacterial microbiota in the human reproductive tract are well described but little is known about the influencing factors. Here, we showed in a relatively large cohort of Caucasian pregnant women that both gestational age as well as reproductive history strongly affect the abundance and prevalence of the dominant vaginal bacteria, many of which have well-established associations to gynaecological and reproductive health. We observed that nulliparity associated strongly with *L. crispatus -*dominated vaginal microbiota in term and late term pregnancies. Prior pregnancies ending in spontaneous or induced abortion did not alter this association. On the other hand, increasing number of prior deliveries associated with decreasing prevalence of *L. crispatus*. Overall, the vaginal microbiota in the late third trimester varied according to the duration of gestation, and especially among women with no previous deliveries, *L. crispatus* was more prevalent with increasing duration of pregnancy.

We found that the vaginal microbiota differed profoundly between nulliparous and multiparous women, indicating that the reproductive history is reflected in the vaginal microbiota at or close to delivery. Our results confirm and extend similar findings reported earlier for the first trimester of pregnancy (between 8- and 12-weeks of gestation),^11^ and for non-pregnant reproductive aged women.^5^ The depletion of vaginal lactobacilli postpartum compared to pregnancy is well established and coincides with the parturition-induced drop in oestrogen levels.^18^^,^^41^ However, the duration of *Lactobacillus*-deficient postpartum microbiota signature has not been thoroughly studied, though there are indications that it remains up to a year postpartum.^42^ Recent research on US-based cohort suggests that such signature is also present in women who delivered by an elective CS,^43^ while another recent study identified birth mode-dependent differences on the vaginal microbiota of Chinese women sampled at comparable timepoints 6 weeks postpartum.^44^ Our preliminary findings also suggest that the mode of delivery might have an impact on the vaginal microbiota as among women with one previous delivery, in those with a history of elective CS, the vaginal microbiota corresponded to that of a nulliparous woman, while after either vaginal delivery or delivery by emergency CS the microbiota diverged. Future studies with proper sample size should specifically address this issue as the results will shed light on the mechanism how previous deliveries leave a lasting effect on the vaginal microbiota.

A successful pregnancy requires tightly coordinated and balanced interplay between host innate immune defences, mucosal immune responses, and the resident microbiota.^45^^,^^46^ Although only vaginal microbiota was determined in the present study, we speculate that the inflammatory and/or adaptive and innate immunity profiles may also differ based on pregnancy history. For instance, immunosuppressive regulatory T cells (FOXP3+ CD4 T lymphocytes), which recognize paternal antigens and are essential for maternal foetal tolerance, accumulate during the first pregnancy and persist to some level in the maternal circulation after delivery.^47^^,^^48^ Later in the subsequent pregnancies these cell populations re-expand in a quicker manner compared with the initial pregnancy.^47^ A pregnancy-induced memory cell response and pregnancy alloimmunization has also been proposed to be behind higher transplantation graft rejection in women with prior pregnancies.^49^

As further support for immunological rather than direct and local microbiological effects, parity has been shown to affect not only the vaginal microbiota but also gut microbiota during subsequent gestation in a pig model.^50^ Hence, our parity-related microbiota findings could reflect the state of the local inflammatory processes and hypothesize some form of an immunological memory from prior labour. This is also supported by our suggestive finding that only previous vaginal delivery or emergency CS, both involving the physiological process of labour, were associated with changes in vaginal microbiota composition. We observed no differences in the relative abundances of *L. crispatus* or *L. gasseri* after elective CS, albeit the limited sample size in this subgroup (n=8) renders our findings merely indicative. Recent studies on healthy non-pregnant, reproductive age women from China^5^ and Belgium^51^ also showed that the vaginal microbiota is significantly associated with past reproductive events, and especially the abundance of *L. crispatus* is decreased in women who have given birth, corroborating our findings.

The ultimate physiological stimuli leading to the onset of labour remain unclear despite intensive research. Although *Lactobacillus*-depleted vaginal microbiota has been associated with increased risk of PTB^52^ studies on microbiota composition at term and especially at late term and prolonged gestation are scarce. The duration of parturition is usually shorter in women with prior deliveries compared to nulliparous^53^ and nulliparity is a known risk factor for prolonged gestation.^54^ We observed that *L. crispatus* dominance increased along the progression of gestational weeks at late pregnancy, especially among nulliparas. This could be explained by higher oestrogen levels at late pregnancy as oestrogen concentrations increase with gestational age,^55^^,^^56^ and due to the higher oestrogen levels during the whole pregnancy among nulliparas than multiparas.^57^ In general, multiparous women without adverse outcomes in previous pregnancies are at lower risk for adverse pregnancy outcomes than nulliparous women.^58^ Hence, our observation of significantly lower abundance of *L. crispatus* in multiparous women compared to nulliparous women contradicts the general recognition of *L. crispatus* vaginal dominance as the hallmark of successful pregnancy outcome. It should, however, be noted that irrespective of the vaginal microbiota composition, all participants in our study delivered a healthy baby at term or late term.

Ripening of the cervix in normal parturition is characterized by inflammatory changes, including the activation of leucocytes and increasing levels of pro-inflammatory cytokines which remodel the extracellular matrix of the cervix.^59^, ^60^, ^61^ An increased ratio of L- to D-lactic acid isomers may alter the cervical tissue integrity by activating matrix metalloprotein inducers.^41^^,^^62^ Low concentrations of D-lactate versus L-lactate have been seen in association with *L. iners* and *G. vaginalis*,^62^ whereas concentrations of D-lactate are higher in *L. crispatus* -dominated vaginal microbiota, reflecting the known metabolic characteristics of vaginal *Lactobacillus* species.^63^ In *in vitro* studies an inflammatory response evoked by *L. crispatus* has been shown to be weaker than by *L. iners* or anaerobic bacteria.^64^ Hence, our findings on the high abundance of *L. crispatus* in late and post term pregnancies may reflect decreased inflammatory bacterial signals. While these findings from a cross-sectional study cannot address causality, it is tempting to hypothesize that the vaginal microbiota and its interactions with the host immune system could play a role in the maintenance of gestation and initiation of spontaneous term or late term labour.

Our results support the data of Romero et al.^65^ who showed in their longitudinal study that the relative abundance of *Lactobacillus* spp., including *L. crispatus*, increased as a function of gestational age. Dissimilar observations have, however, been presented as Avershina et al.^20^ first reported an increasing diversity of the vaginal microbiota at the onset of labour compared to samples taken at 36 weeks of gestation. Later, Rasmussen et al.^19^ observed that there was a gradual decline of *Lactobacillus* spp. from week 24 of pregnancy until birth while only genera *Enterococcus* and *Granulicatella* were associated with gestational age at birth. Their sample size (n=57) was small compared to ours and the last samples were taken later during birth after rupture of membranes so the results might not be thoroughly comparable. Furthermore, the proportion of nulliparous women, in whom the association between higher gestational age and *L. crispatus* dominance was more pronounced in our study, was lower in their study (40% vs 58%).

Identifying women susceptible to prolongation of pregnancy and its associated complications would be of great clinical benefit. Prolonged pregnancy increases the risk of stillbirth, neonatal morbidity and mortality, and the risk of CS associated to the induction of labour is higher than in inductions in earlier pregnancy weeks.^66^ Nulliparity is one of the known risk factors for prolonged pregnancy,^67^ and nulliparas form the majority of women undergoing induction of labour due to prolonged pregnancy^26^ Previously *Lactobacillus* abundances have been shown to decline gradually toward birth.^19^ Prevalence of *L. crispatus* -dominated vaginal microbiota, however, was higher after the due date among nulliparas in our study. Although this finding alone is not sufficient to predict the duration of gestation, more detailed research in inflammatory and immunological pathway activation in addition to the microbiota composition could well help to identify women at risk of prolonged gestation. Also, as different clinical scoring systems to predict the success of labour induction have been proposed,^68^ we may speculate that the vaginal microbiota could potentially be included in the evaluation to decrease the rates of unsuccessful labour inductions which could decrease maternal and neonatal complications and affect maternal birth experience.

The strengths of our study include the prospective setting and relatively high number of study subjects and the use of well-established sequencing techniques for analysing the microbiota. The study population was homogenic with comprehensive outcome and background information from medical records and the specific questionnaire designed for the study. To our knowledge, this study is the first to characterize the vaginal microbiota at late pregnancy stages in a large cohort of women with comprehensive obstetric records. The main limitation in our study is sampling at a single timepoint for each participant. A longitudinal study with samples from early pregnancy until delivery would allow to see the potential intraindividual changes in the microbiota with advancing gestation. The homogeneity of our study population, despite it being also an asset, does not let us interpret whether the results can be generalized to women with different ethnic and biogeographical backgrounds knowing that ethnicity-related differences in the vaginal microbiota exist also during pregnancy.^41^^,^^69^ A larger international multicentre study instead of a single centre study like ours would be needed to show whether these results can be generalized. There might also be residual confounding due to self-reporting of e.g. sexual habits and education. Another limitation in our study is that a variety of other variables unrelated to pregnancy history or gestational age, such as genetics, diet, stress, and viral infections, can also have an influence on vaginal microbiota composition^70^ and these could not be acknowledged in our study.

In conclusion, previous pregnancy history has a strong association with the composition of vaginal microbiota close to parturition, while among nulliparous women, gestational age associates with the microbiota. The findings are intriguing, agree with previous publications, and extend their findings of the few currently known factors associated with the duration of gestation (e.g. the relationship of maternal age, nulliparity, and obesity to post term pregnancy).^54^ Whether the microbiota changes just reflect or actively contribute to the underlying immunological processes and mechanisms remain to be studied. As the vaginal microbiota is, at least conceptually, modifiable, our findings highlight the importance of future studies to understand the nature of implicated host-microbiota interactions and to investigate its potential for diagnostic and therapeutic approaches. Pregnancy history and duration of gestation at the time of sample collection must be acknowledged in future studies on vaginal microbiota.

## Contributors

KK, IK, AS and PN conceptualized and designed the study. KK and SS analysed and verified all underlying data. Patient recruitment, enrolment, and sample collection: KK, IK, TH. Analysis and interpretation of data: KK, SS, TH, SV, AS, IK. Writing the first version of the manuscript: KK, TH. Writing, review, and revision of the manuscript: KK, SS, IK, TH, SV, LR, VS, AS, PN. All authors read, edited, revised, approved the final version of the manuscript, and were responsible for the decision to submit the manuscript. These authors contributed equally: Tiina Holster and Schahzad Saqib; Anne Salonen and Ilkka Kalliala.

## Data sharing statement

The sequencing data generated in this study have been deposited at the European Nucleotide Archive (ENA) with the project accession number PRJEB47492. The code scripts used for data processing, analysis, and visualization have been deposited at GitHub (https://github.com/SchahzadSaqib/EMV) and are publicly available as of the date of publication. Any additional information required to reanalyse the data reported in this paper is available from the corresponding author upon request.

## Declaration of interests

The authors declare no competing interests. KK has received doctoral student salary from the University of Helsinki, and grants from Orion Research Foundation and the Finnish Medical Foundation. VS and LR have received clinical researcher grants. AS has received a grant from the European Union's Horizon 2020 research and innovation programme, and article processing charges from the University of Helsinki. IK has received grants from the Academy of Finland, the Finnish Medical Foundation, and State Research Funding. VS is a board member of the International Society for Placenta Accreta Spectrum, and IK is a board member of the Finnish Colposcopy Society.

## Appendix Supplementary materials

Image, application 1
