# Supplementary material for: Parity and gestational age are associated with vaginal microbiota composition in term and late term pregnancies
Source: eBioMedicine. 2022 Jun 24;81:104107. doi: 10.1016/j.ebiom.2022.104107 (PMC9250009; doi:10.1016/j.ebiom.2022.104107)
Supplement: Supplementary file 1 [file mmc1.pdf]

Supplementary Information

Parity and gestational age are associated with vaginal microbiota composition in term and late term pregnancies

Kaisa Kervinen[1,2], Tiina Holster[1§], Schahzad Saqib[2§], Seppo Virtanen[1,2], Vedran Stefanovic[1], Leena Rahkonen[1], Pekka Nieminen[1], Anne Salonen[2*#], Ilkka Kalliala[1,2,3#]

[1] Department of Obstetrics and Gynaecology, University of Helsinki and Helsinki University Hospital, Helsinki, Finland
[2] Human Microbiome Research Program, Faculty of Medicine, University of Helsinki, Helsinki, Finland
[3] Department of Metabolism, Digestion and Reproduction, Faculty of Medicine, Imperial College London, London, UK

[*] Corresponding author E-mail: anne.salonen@helsinki.fi
[§] and [#] Contributed equally

Table of Contents

Figures 3

Supplementary Fig. 1. Phylogenetic tree. 3

Supplementary Fig. 2: Parity. 4

Supplementary Fig. 3: Previous delivery method. 5

Supplementary Fig. 4: Parity (3 class). 5

Data Tables 6

Supplementary Table 1 | Characteristics of the study population 6

Taxonomic distributions 7

Supplementary Table 2 | Genus detection >5% 7

Supplementary Table 3 | Genus dominance >5% 7

Supplementary Table 4 | Species detection > 5% (top) 8

Supplementary Table 5 | Species dominance >50% (top) 9

Background variables 10

Supplementary Table 6 | Associations between individual background variables and the overall microbiota variation (n=324) 10

Statistics 11

Supplementary Table 7 | Model Summaries – Parity related variables 11

Supplementary Table 8 | Model Summaries – Parity related variables adjusted for age 13

Supplementary Table 9 | Model Summaries – Gestational age-related variables 14

Supplementary Table 10 | Model Summaries – Gestational age-related variables adjusted for age, BMI, and smoking 16

Supplementary Table 11 | Model Summaries – Demographic background variables 17

Figures

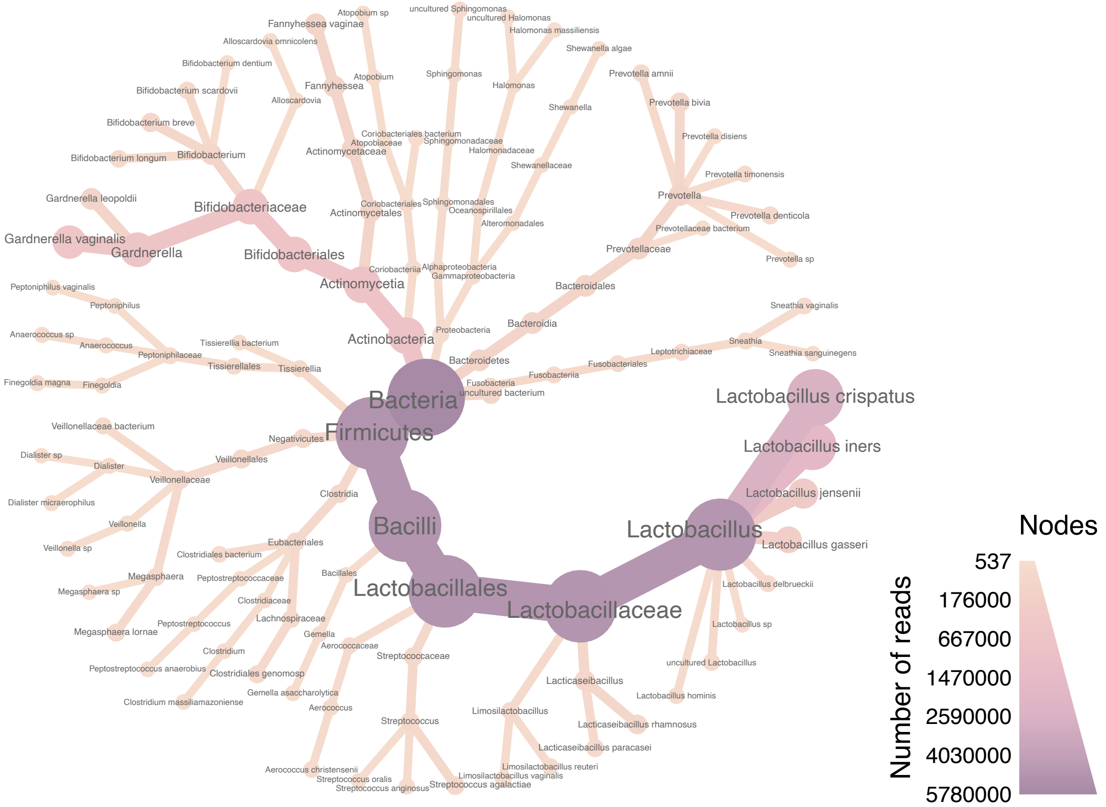

Supplementary Fig. 1. Phylogenetic tree.
A phylogenetic tree of bacterial species identified from the 16S rRNA gene sequencing data across all (n=324) samples.

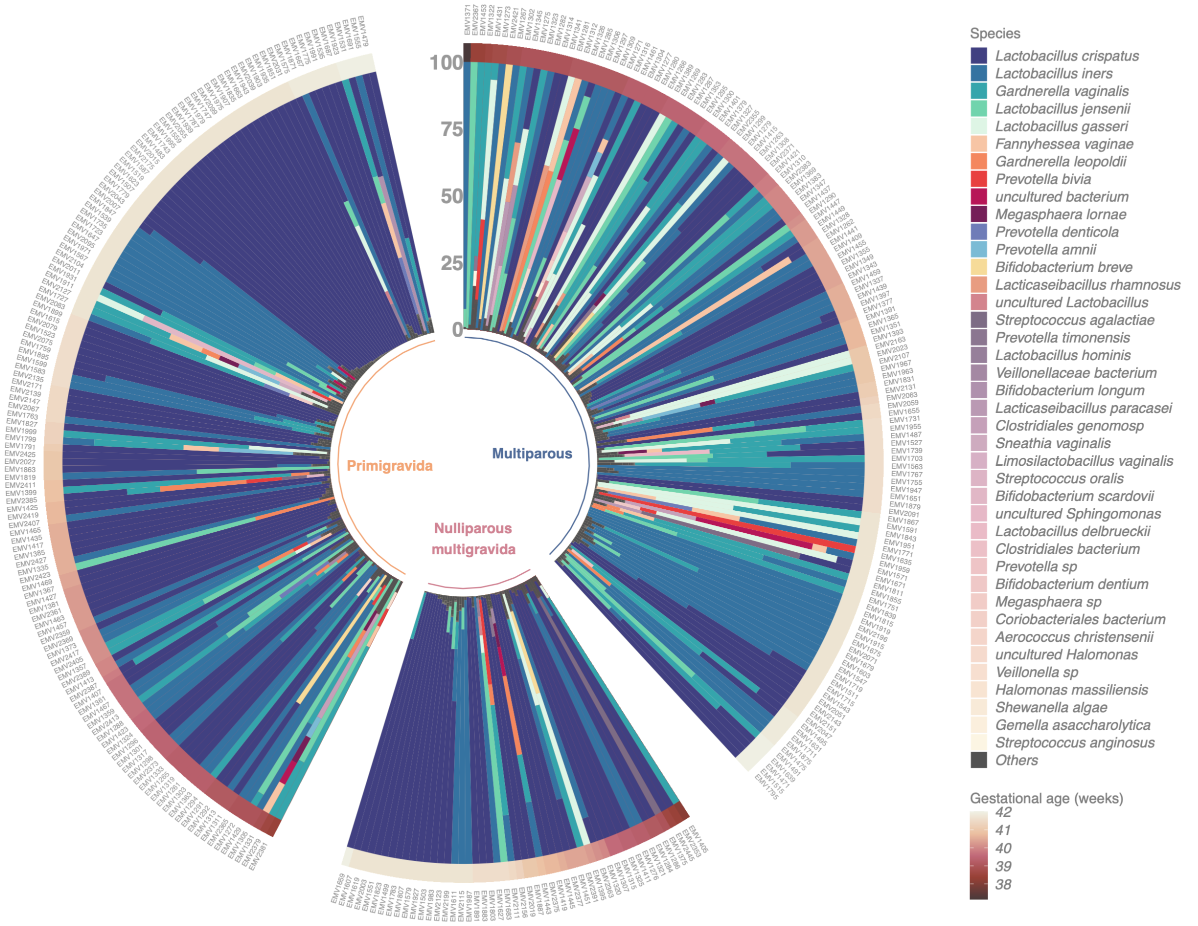

Supplementary Fig. 2: Parity.
Circular stacked bar chart showing the bacterial relative abundances of all the samples in the study cohort (n=324), divided based on parity – no previous pregnancies or deliveries (nulliparous primigravida) (n=139), previous pregnancies but no deliveries (nulliparous non-primigravida) (n=49) and one or more previous deliveries (multiparous) (n=136). The samples have been ordered based on two criteria 1) gestational age in weeks, increasing clockwise and 2) within each gestational age bracket, whether one of the top 3 bacteria were the most abundant (>50% composition), ordered clockwise from None, to *Gardnerella vaginalis*, *Lactobacillus iners*, and *Lactobacillus crispatus* dominant samples.

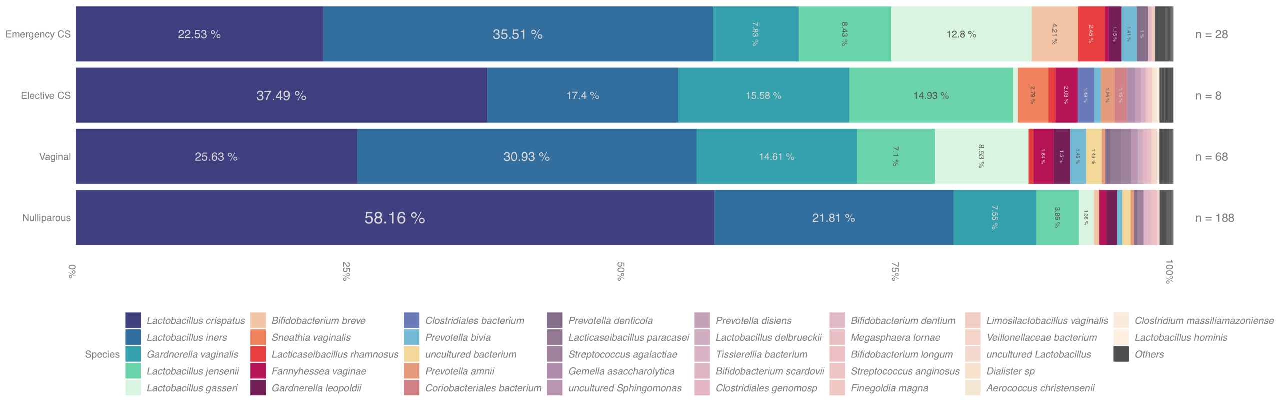

Supplementary Fig. 3: Previous delivery method.
Stacked bar plot depicting the mean bacterial relative abundances of samples from women without prior pregnancies or deliveries (nulliparous) (n=188) and women with one prior delivery in history (n=104) divided by delivery method.

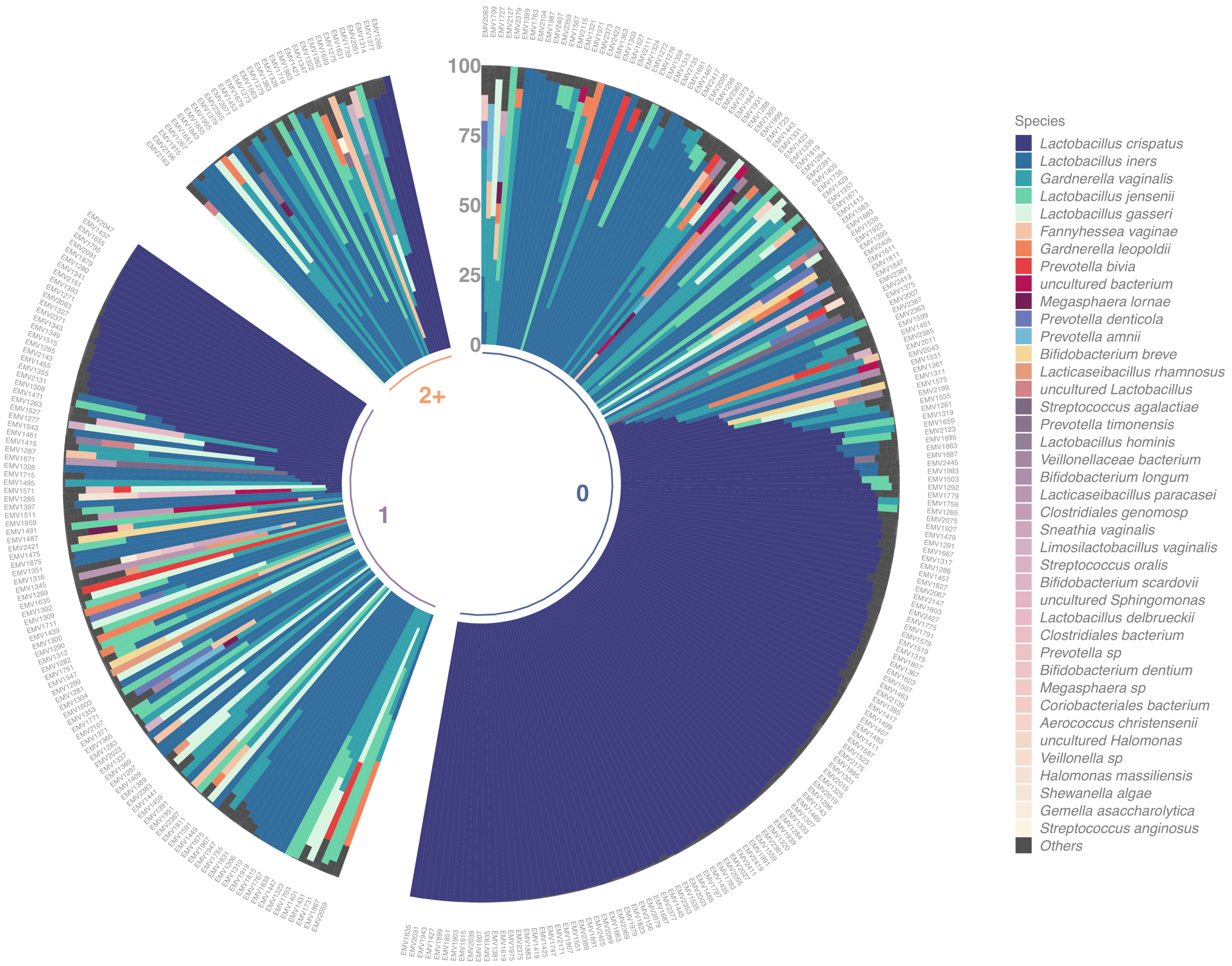

Supplementary Fig. 4: Parity (3 class).
Circular stacked bar chart showing the bacterial relative abundances of all the samples in the study cohort (n=324), divided based on parity – 0 previous deliveries (n=188), 1 previous delivery (n=104) and 2 or more previous deliveries (n=32).

Data Tables

Supplementary Table 1 | Characteristics of the study population

Characteristics	Elective CS	Full term birth	Late term pregnancy	*p*-value
Number of women	60	96	168	
Age, years				
Mean (SD, range)[1]	34·5 (4·46, 21–47)	30·2 (4·10, 20–39)	32·3 (4·90, 20–43)	**<0·0001**
BMI, kg/m[2]				
Median (IQR)[2]	23·9 (22·0–26·0)	22·2 (20·6–24·2)	24·0 (21·5–27·4)	**0·0014**
Nullipara[3]	25/60 (41·7)	58/96 (60·4)	105/168 (62·5)	**0·017**
Primigravida	17/25 (68·0)	44/58 (75·9)	78/105 (74·3)	0·75
Nulliparous multigravida	8/25 (32·0)	14/58 (24·1)	27/105 (25·7)	
Current smoking[3]	3/60 (5·0)	4/96 (4·2)	3/168 (1·8)	0·30
Infertility treatments (current pregnancy) [3]	7/60 (11·7)	9/96 (9·4)	6/168 (3·6)	**0·049**
Use of probiotics[*, 3]	39/54 (72·2)	59/88 (56·8)	103/161 (64·0)	0·18
Antibiotic use (<3 months)[*, 3]	5/53 (9·4)	11/88 (12·5)	29/161 (18·0)	0·24
Intercourse <48h[*, 3]	3/54 (5·6)	12/89 (13·5)	31/161 (19·3)	**0·046**
High education (tertiary degree)[*, 3]	28/54 (70·4)	54/86 (62·8)	106/163 (65·0)	0·65
Married or cohabiting[*, 3]	59/54 (92·6)	84/86 (97·7)	154/164 (93·9)	0·30
Pregnancy related characteristics				
Gestational age at sampling, weeks				
Median (IQR)[2]	39·3 (39·1–39·5)	40·1 (39·6–40·6)	41·7 (41·6–41·7)	**<0·0001**
Gestational age at birth, weeks				
Median (IQR)[2]	39·3 (39·1–39·5)	40·3 (39·6–40·6)	41·9 (41·9–42·0)	**<0·0001**
Gestational diabetes[3]	15/60 (25·0)	22/96 (22·9)	42/168 (25·0)	0·92
Maternal postpartum infection[†, 3]	3/60 (5·0)	8/96 (8·3)	21/168 (12·5)	0·21
Birthweight, g				
Mean (SD, range)[1]	3547 (426, 2732–4730)	3531 (418, 2636–4744)	3811 (482, 2740–5130)	**<0·0001**

CS, caesarean section; BMI, body mass index; SD, standard deviation; IQR, interquartile range
Data are n (%) unless otherwise specified.

*Missing information for data collected from questionnaires, total number of women for which data is known is shown for each variable.

†Postpartum infections included endometritis n=24, episiotomy infection n=6, urinary tract infection n=1, post-caesarean section wound infection n=1.

[1] ANOVA

[2] Kruskal-Wallis test

[3] Chi square and Fisher's exact test when appropriate

Taxonomic distributions

Supplementary Table 2 | Genus detection >5%

genus	genus_tally	genus_tally_prc
Lactobacillus	309	95·4%
Gardnerella	81	25·0%
Prevotella	23	7·10%
Fannyhessea	18	5·60%
uncultured	11	3·40%
Bifidobacterium	8	2·50%
Megasphaera	8	2·50%
Lacticaseibacillus	5	1·50%
Streptococcus	5	1·50%
Veillonellaceae	3	0·90%
Clostridiales	3	0·90%
Limosilactobacillus	2	0·60%
Sneathia	2	0·60%
Halomonas	1	0·30%
Shewanella	1	0·30%
Aerococcus	1	0·30%
Coriobacteriales	1	0·30%
Gemella	1	0·30%
Veillonella	1	0·30%

Supplementary Table 3 | Genus dominance >5%

genus	genus_tally	genus_tally_prc
Lactobacillus	256	79·0%
Gardnerella	30	9·30%
Bifidobacterium	4	1·20%
Streptococcus	2	0·60%
Fannyhessea	1	0·30%
Lacticaseibacillus	1	0·30%
Prevotella	1	0·30%

Supplementary Table 4 | Species detection > 5% (top)

Sample subgroup	Bacteria	Detection tally	Subgroup tally	%age
Pooled	*Lactobacillus crispatus*	170	324	52·50%
Nulliparous	*Lactobacillus crispatus*	123	188	65·40%
Primigravida	*Lactobacillus crispatus*	87	139	62·60%
Multiparous	*Lactobacillus crispatus*	47	136	34·60%
Nulliparous multigravida	*Lactobacillus crispatus*	36	49	73·50%
Pooled	*Lactobacillus iners*	119	324	36·70%
Multiparous	*Lactobacillus iners*	63	136	46·30%
Nulliparous	*Lactobacillus iners*	56	188	29·80%
Primigravida	*Lactobacillus iners*	43	139	30·90%
Nulliparous multigravida	*Lactobacillus iners*	13	49	26·50%
Pooled	*Gardnerella vaginalis*	78	324	24·10%
Multiparous	*Gardnerella vaginalis*	40	136	29·40%
Nulliparous	*Gardnerella vaginalis*	38	188	20·20%
Primigravida	*Gardnerella vaginalis*	30	139	21·60%
Nulliparous multigravida	*Gardnerella vaginalis*	8	49	16·30%
Pooled	*Lactobacillus jensenii*	48	324	14·80%
Multiparous	*Lactobacillus jensenii*	25	136	18·40%
Nulliparous	*Lactobacillus jensenii*	23	188	12·20%
Primigravida	*Lactobacillus jensenii*	19	139	13·70%
Nulliparous multigravida	*Lactobacillus jensenii*	4	49	8·20%
Pooled	*Lactobacillus gasseri*	31	324	9·60%
Multiparous	*Lactobacillus gasseri*	21	136	15·40%
Nulliparous	*Lactobacillus gasseri*	10	188	5·30%
Primigravida	*Lactobacillus gasseri*	6	139	4·30%
Nulliparous multigravida	*Lactobacillus gasseri*	4	49	8·20%
Pooled	*Fannyhessea vaginae*	18	324	5·60%
Multiparous	*Fannyhessea vaginae*	10	136	7·40%
Nulliparous	*Fannyhessea vaginae*	8	188	4·30%
Primigravida	*Fannyhessea vaginae*	8	139	5·80%
Pooled	*Gardnerella leopoldii*	18	324	5·60%
Nulliparous	*Gardnerella leopoldii*	11	188	5·90%
Primigravida	*Gardnerella leopoldii*	9	139	6·50%
Multiparous	*Gardnerella leopoldii*	7	136	5·10%
Nulliparous multigravida	*Gardnerella leopoldii*	2	49	4·10%

Supplementary Table 5 | Species dominance >50% (top)

Sample subgroup	Bacteria	Dominance tally	Subgroup tally	%age
Pooled	*Lactobacillus crispatus*	142	324	43·80%
Nulliparous	*Lactobacillus crispatus*	111	188	59·00%
Primigravida	*Lactobacillus crispatus*	77	139	55·40%
Nulliparous multigravida	*Lactobacillus crispatus*	34	49	69·40%
Multiparous	*Lactobacillus crispatus*	31	136	22·80%
Pooled	*Lactobacillus iners*	90	324	27·80%
Multiparous	*Lactobacillus iners*	46	136	33·80%
Nulliparous	*Lactobacillus iners*	44	188	23·40%
Primigravida	*Lactobacillus iners*	36	139	25·90%
Nulliparous multigravida	*Lactobacillus iners*	8	49	16·30%
Pooled	*Gardnerella vaginalis*	29	324	9·0%
Multiparous	*Gardnerella vaginalis*	19	136	14·0%
Nulliparous	*Gardnerella vaginalis*	10	188	5·30%
Primigravida	*Gardnerella vaginalis*	8	139	5·80%
Nulliparous multigravida	*Gardnerella vaginalis*	2	49	4·10%
Pooled	*Lactobacillus jensenii*	12	324	3·70%
Multiparous	*Lactobacillus jensenii*	8	136	5·90%
Nulliparous	*Lactobacillus jensenii*	4	188	2·10%
Primigravida	*Lactobacillus jensenii*	3	139	2·20%
Nulliparous multigravida	*Lactobacillus jensenii*	1	49	2·0%
Pooled	*Lactobacillus gasseri*	12	324	3·70%
Multiparous	*Lactobacillus gasseri*	11	136	8·10%
Nulliparous	*Lactobacillus gasseri*	1	188	0·50%
Nulliparous multigravida	*Lactobacillus gasseri*	1	49	2·0%

Background variables

Supplementary Table 6 | Associations between individual background variables and the overall microbiota variation (n=324)

Characteristics	Df	F value	R2	*p*-value
Age, years	1	0·46	0·001	0·74
BMI, kg/m^2	1	0·88	0·003	0·43
Nulliparity (yes vs. no)	1	21·30	0·062	***1·00E-05***
Current smoking (yes vs. no)	1	2·25	0·007	0·076
History of smoking (yes vs. no)	1	2·24	0·007	0·077
Use of probiotics (yes vs.no)	1	0·49	0·002	0·72
Antibiotics in recent 3 months (yes vs. no)	1	2·25	0·007	0·075
Intercourse <48 hours prior to sample (yes vs. no)	1	1·56	0·005	0·18
Level of education (university or polytechnic vs. no)	1	4·38	0·014	***6·03E-03***
Level of education (5 class)	4	2·50	0·032	***3·73E-03***
Sex partners during lifetime (over 3 vs. less)	1	3·73	0·013	***1·36E-02***
Married or cohabiting (yes vs. no)	1	0·62	0·002	0·61
Infertility treatments before current pregnancy (yes vs. no)	1	0·70	0·002	0·55
Infertility treatments ever (yes vs. no)	1	0·76	0·003	0·51
**Pregnancy related characteristics**				
Gestational age at sample, weeks	1	4·44	0·014	***6·72E-03***
Gestational age at birth, weeks	1	4·94	0·015	***4·05E-03***
Contractions at sampling (yes vs.no)	1	0·83	0·003	0·46
Sampling group (elective CS, during labour, late-term or prolonged)	2	1·23	0·008	0·27
Gestational diabetes (yes vs. no)	1	0·48	0·001	0·72
Maternal postpartum infection (yes vs. no)	1	0·87	0·003	0·43
Neonatal birthweight, g	1	0·40	0·001	0·79
**Technical variables**				
Sequencing run	3	1·15	0·011	0·30
Sample weight	1	1·02	0·003	0·36
Read count	1	2·56	0·007	0·050
DNA concentration	1	2·60	0·008	0·05

Associations between individual background variables and the microbiota were analysed with permutational ANOVA with 99,999 permutations.

Statistics

Supplementary Table 7 | Model Summaries – Parity related variables

	Fannyhessea vaginae	*Gardnerella leopoldii*	*Gardnerella vaginalis*	*Lactobacillus crispatus*	*Lactobacillus gasseri*	*Lactobacillus iners*	*Lactobacillus jensenii*
**Nulliparous (Reference) vs. Multiparous**							
Mean (Multiparous)	9·805E-03	7·096E-03	1·378E-01	2·329E-01	6·179E-02	3·345E-01	6·492E-02
Mean (Nulliparous)	4·847E-03	6·339E-03	7·335E-02	5·816E-01	1·245E-02	2·181E-01	3·143E-02
FoldChange	2·023E+00	1·119E+00	1·878E+00	-2·498E+00	4·963E+00	1·534E+00	2·065E+00
Estimate	7·048E-01	1·132E-01	6·846E-01	-2·337E+00	1·598E+00	1·050E+00	7·253E-01
Estimate FoldChange	2·023E+00	1·120E+00	1·983E+00	9·662E-02	4·942E+00	2·858E+00	2·065E+00
p.values	2·432E-01	6·710E-01	9·873E-02	2·496E-09	2·100E-08	1·245E-02	6·247E-02
FDR corrected p.values (q.values)	2·837E-01	6·710E-01	1·382E-01	**1·747E-08**	**7·351E-08**	**2·906E-02**	1·093E-01
Model	MASS - glm.nb	MASS - glm.nb	log nlme - gls	log nlme - gls	MASS - glm.nb	log nlme - gls	MASS - glm.nb
**Primigravida (Reference) vs. Multiparous & Nulliparous multigravida**							
Mean (Multiparous)	9·805E-03	7·096E-03	1·378E-01	2·329E-01	6·179E-02	3·345E-01	6·492E-02
Mean (Nulliparous multigravida)	7·974E-04	5·160E-03	5·244E-02	6·645E-01	1·974E-02	1·597E-01	2·128E-02
Mean (Primigravida)	6·275E-03	6·755E-03	8·072E-02	5·524E-01	9·879E-03	2·386E-01	3·501E-02
FoldChange (Multiparous)	1·563E+00	1·051E+00	1·707E+00	-4·216E-01	6·254E+00	1·402E+00	1·854E+00
FoldChange (Nulliparous multigravida)	-1·271E-01	-7·638E-01	-6·497E-01	1·203E+00	1·998E+00	-6·692E-01	-6·079E-01
Estimate (Multiparous)	4·466E-01	5·002E-02	6·502E-01	-2·107E+00	1·828E+00	7·745E-01	6·175E-01
Estimate (Nulliparous multigravida)	-2·065E+00	-2·676E-01	-1·320E-01	8·835E-01	6·890E-01	-1·057E+00	-4·978E-01
Estimate FoldChange (Multiparous)	1·563E+00	1·051E+00	1·916E+00	1·216E-01	6·220E+00	2·170E+00	1·854E+00
Estimate FoldChange (Nulliparous multigravida)	1·269E-01	7·652E-01	8·764E-01	2·419E+00	1·992E+00	3·476E-01	6·079E-01
p.values (vs. Multiparous)	4·839E-01	8·608E-01	1·409E-01	4·447E-07	1·993E-09	8·256E-02	1·383E-01
p.values (vs. Nulliparous multigravida)	1·892E-02	4·959E-01	8·121E-01	1·107E-01	1·007E-01	8·281E-02	3·858E-01
FDR corrected p.values (q.values, vs. Multiparous)	5·646E-01	8·608E-01	1·973E-01	**1·556E-06**	**1·395E-08**	1·926E-01	1·973E-01
FDR corrected p.values (q.values, vs. Nulliparous multigravida)	1·324E-01	5·786E-01	8·121E-01	1·937E-01	1·937E-01	1·937E-01	5·401E-01
Model	MASS - glm.nb	MASS - glm.nb	log nlme - gls	log nlme - gls	MASS - glm.nb	log nlme - gls	MASS - glm.nb
**Nulliparous multigravida (Reference) vs. Multiparous & Primigravida**							
Mean (Multiparous)	9·805E-03	7·096E-03	1·378E-01	2·329E-01	6·179E-02	3·345E-01	6·492E-02
Mean (Nulliparous multigravida)	7·974E-04	5·160E-03	5·244E-02	6·645E-01	1·974E-02	1·597E-01	2·128E-02
Mean (Primigravida)	6·275E-03	6·755E-03	8·072E-02	5·524E-01	9·879E-03	2·386E-01	3·501E-02
FoldChange (Multiparous)	1·230E+01	1·375E+00	2·627E+00	-2·854E+00	3·130E+00	2·095E+00	3·050E+00

FoldChange (Primigravida)	7·870E+00	1·309E+00	1·539E+00	-1·203E+00	-1·998E+00	1·494E+00	1·645E+00
Estimate (Multiparous)	2·511E+00	3·177E-01	7·821E-01	-2·990E+00	1·139E+00	1·831E+00	1·115E+00
Estimate (Primigravida)	2·065E+00	2·676E-01	1·320E-01	-8·835E-01	-6·890E-01	1·057E+00	4·978E-01
Estimate FoldChange (Multiparous)	1·232E+01	1·374E+00	2·186E+00	5·028E-02	3·123E+00	6·242E+00	3·051E+00
Estimate FoldChange (Primigravida)	7·882E+00	1·307E+00	1·141E+00	4·133E-01	5·021E-01	2·877E+00	1·645E+00
p.values (vs. Multiparous)	4·418E-03	4·203E-01	1·775E-01	1·561E-07	6·819E-03	3·129E-03	5·266E-02
p.values (vs. Primigravida)	1·892E-02	4·959E-01	8·121E-01	1·107E-01	1·007E-01	8·281E-02	3·858E-01
FDR corrected p.values (q.values, vs. Multiparous)	**1·031E-02**	4·203E-01	2·071E-01	**1·092E-06**	**1·193E-02**	**1·031E-02**	7·372E-02
FDR corrected p.values (q.values, vs. Primigravida)	1·324E-01	5·786E-01	8·121E-01	1·937E-01	1·937E-01	1·937E-01	5·401E-01
Model	MASS - glm.nb	MASS - glm.nb	log nlme - gls	log nlme - gls	MASS - glm.nb	log nlme - gls	MASS - glm.nb
**Previous birth mode: Nulliparous, n=188 (Reference) vs. 1 delivery, n=104 (Elective CS)**							
Mean (Elective CS)	2·031E-02	4·026E-04	1·558E-01	3·749E-01	4·269E-03	1·740E-01	1·212E-01
Mean (Nulliparous)	4·715E-03	6·346E-03	7·180E-02	5·816E-01	1·205E-02	2·181E-01	3·103E-02
FoldChange	4·307E+00	-1·576E+01	2·169E+00	-1·552E+00	-2·822E+00	-1·253E+00	3·905E+00
Estimate	9·673E-01	-2·665E+00	4·468E-01	-1·623E+00	-1·034E+00	-2·257E-01	1·362E+00
Estimate FoldChange	2·631E+00	6·960E-02	1·563E+00	1·974E-01	3·557E-01	7·979E-01	3·906E+00
p.values	4·282E-01	1·785E-03	7·337E-01	1·967E-01	2·545E-01	7·799E-01	2·666E-01
FDR corrected p.values (q.values)	5·995E-01	**1·250E-02**	7·799E-01	4·665E-01	4·665E-01	7·799E-01	4·665E-01
Model	log nlme - gls	MASS - glm.nb	log nlme - gls	log nlme - gls	MASS - glm.nb	MASS - glm.nb	MASS - glm.nb
**Previous birth mode: Nulliparous, n=188 (Reference) vs. 1 delivery, n=104 (Emergency CS)**							
Mean (Emergency CS)	3·541E-03	4·385E-03	7·768E-02	2·253E-01	1·049E-01	3·551E-01	6·915E-02
Mean (Nulliparous)	4·715E-03	6·346E-03	7·180E-02	5·816E-01	1·205E-02	2·181E-01	3·103E-02
FoldChange	-1·332E+00	-1·447E+00	1·082E+00	-2·582E+00	8·705E+00	1·628E+00	2·228E+00
Estimate	-4·081E-02	-3·695E-01	-3·012E-01	-2·693E+00	2·159E+00	4·874E-01	8·012E-01
Estimate FoldChange	9·600E-01	6·911E-01	7·399E-01	6·765E-02	8·660E+00	1·628E+00	2·228E+00
p.values	9·277E-01	4·370E-01	6·572E-01	1·879E-04	2·195E-05	2·823E-01	2·443E-01
FDR corrected p.values (q.values)	9·277E-01	6·118E-01	7·667E-01	**6·578E-04**	**1·536E-04**	4·940E-01	4·940E-01
Model	log nlme - gls	MASS - glm.nb	log nlme - gls	log nlme - gls	MASS - glm.nb	MASS - glm.nb	MASS - glm.nb
**Previous birth mode: Nulliparous, n=188 (Reference) vs. 1 delivery, n=104 (Vaginal delivery)**							
Mean (Vaginal delivery)	1·074E-02	7·200E-03	1·372E-01	2·563E-01	5·465E-02	3·093E-01	5·466E-02
Mean (Nulliparous)	4·715E-03	6·346E-03	7·180E-02	5·816E-01	1·205E-02	2·181E-01	3·103E-02
FoldChange	2·278E+00	1·135E+00	1·911E+00	-2·270E+00	4·537E+00	1·418E+00	1·761E+00
Estimate	7·385E-01	1·290E-01	8·376E-01	-2·231E+00	1·508E+00	3·494E-01	5·661E-01
Estimate FoldChange	2·093E+00	1·138E+00	2·311E+00	1·074E-01	4·519E+00	1·418E+00	1·761E+00

p.values	3·374E-02	6·976E-01	1·131E-01	8·565E-06	2·192E-05	2·699E-01	2·390E-01
FDR corrected p.values (q.values)	7·872E-02	6·976E-01	1·978E-01	**5·995E-05**	**7·671E-05**	3·149E-01	3·149E-01
Model	log nlme - gls	MASS - glm.nb	log nlme - gls	log nlme - gls	MASS - glm.nb	MASS - glm.nb	MASS - glm.nb
**Parity**							
Estimate	3·581E-01	8·586E-02	5·226E-01	-1·084E+00	9·098E-01	5·881E-01	3·586E-01
p.values	2·935E-01	5·614E-01	3·863E-02	2·059E-06	2·144E-08	1·955E-02	1·032E-01
FDR corrected p.values (q.values)	3·424E-01	5·614E-01	6·761E-02	**7·206E-06**	**1·501E-07**	**4·562E-02**	1·444E-01
Model	MASS - glm.nb	nlme - gls	nlme - gls	nlme - gls	MASS - glm.nb	nlme - gls	MASS - glm.nb
**Time (months) since previous delivery and sample, n=111**							
Estimate	-1·594E-02	-6·666E-03	-3·312E-02	8·966E-03	-1·351E-03	4·866E-03	1·187E-02
p.values	2·469E-02	3·957E-01	5·617E-03	3·741E-01	9·234E-01	7·504E-01	4·237E-01
FDR corrected p.values (q.values)	8·640E-02	5·931E-01	**3·932E-02**	5·931E-01	9·234E-01	8·754E-01	5·931E-01
Model	nlme - gls	nlme - gls	nlme - gls	MASS - glm.nb	nlme - gls	nlme - gls	MASS - glm.nb

Supplementary Table 8 | Model Summaries – Parity related variables adjusted for age

	Fannyhessea vaginae	***Gardnerella leopoldii***	***Gardnerella vaginalis***	***Lactobacillus crispatus***	***Lactobacillus gasseri***	***Lactobacillus iners***	***Lactobacillus jensenii***
**Nulliparous (Reference) vs. Multiparous**							
Estimate	6·381E-01	8·776E-02	7·271E-01	-2·375E+00	1·578E+00	1·152E+00	6·751E-01
Estimate FoldChange	1·751E+00	1·092E+00	1·945E+00	9·664E-02	4·845E+00	2·864E+00	1·964E+00
p.values	1·524E-02	7·429E-01	7·985E-02	2·047E-09	4·107E-08	6·596E-03	8·569E-02
FDR corrected p.values (q.values)	**2·668E-02**	7·429E-01	9·997E-02	**1·433E-08**	**1·437E-07**	**1·539E-02**	9·997E-02
Model	log nlme - gls	MASS - glm.nb	log nlme - gls	log nlme - gls	MASS - glm.nb	log nlme - gls	MASS - glm.nb
**Primigravida (Reference) vs. Multiparous & Nulliparous multigravida**							
Estimate (Multiparous)	4·454E-01	9·144E-02	7·404E-01	-2·145E+00	1·833E+00	9·058E-01	5·239E-01
Estimate (Nulliparous multigravida)	-1·857E+00	1·452E-02	4·412E-02	8·349E-01	7·921E-01	-8·897E-01	-5·352E-01
Estimate FoldChange (Multiparous)	1·561E+00	1·096E+00	1·911E+00	1·216E-01	6·253E+00	2·180E+00	1·689E+00
Estimate FoldChange (Nulliparous multigravida)	1·561E-01	1·015E+00	9·266E-01	2·417E+00	2·208E+00	3·485E-01	5·856E-01
p.values (vs. Multiparous)	4·912E-01	7·519E-01	9·707E-02	4·556E-07	3·329E-09	4·593E-02	2·166E-01
p.values (vs. Nulliparous multigravida)	3·688E-02	9·708E-01	9·387E-01	1·385E-01	6·286E-02	1·486E-01	3·584E-01
FDR corrected p.values (q.values, vs. Multiparous)	5·731E-01	7·519E-01	1·699E-01	**1·594E-06**	**2·330E-08**	1·072E-01	3·033E-01
FDR corrected p.values (q.values, vs. Nulliparous multigravida)	2·200E-01	9·708E-01	9·708E-01	2·600E-01	2·200E-01	2·600E-01	5·017E-01

Model	MASS - glm.nb	MASS - glm.nb	log nlme - gls	log nlme - gls	MASS - glm.nb	log nlme - gls	MASS - glm.nb
**Nulliparous multigravida (Reference) vs. Multiparous & Primigravida**							
Estimate (Multiparous)	2·303E+00	7·692E-02	6·963E-01	-2·980E+00	1·041E+00	1·796E+00	1·059E+00
Estimate (Primigravida)	1·857E+00	-1·452E-02	-4·412E-02	-8·349E-01	-7·921E-01	8·897E-01	5·352E-01
Estimate FoldChange (Multiparous)	1·000E+01	1·080E+00	2·062E+00	5·030E-02	2·832E+00	6·256E+00	2·884E+00
Estimate FoldChange (Primigravida)	6·406E+00	9·856E-01	1·079E+00	4·137E-01	4·529E-01	2·870E+00	1·708E+00
p.values (vs. Multiparous)	8·842E-03	8·447E-01	2·334E-01	1·832E-07	1·336E-02	3·770E-03	6·589E-02
p.values (vs. Primigravida)	3·688E-02	9·708E-01	9·387E-01	1·385E-01	6·286E-02	1·486E-01	3·584E-01
FDR corrected p.values (q.values, vs. Multiparous)	**2·063E-02**	8·447E-01	2·723E-01	**1·282E-06**	**2·339E-02**	**1·320E-02**	9·225E-02
FDR corrected p.values (q.values, vs. Primigravida)	2·200E-01	9·708E-01	9·708E-01	2·600E-01	2·200E-01	2·600E-01	5·017E-01
Model	MASS - glm.nb	MASS - glm.nb	log nlme - gls	log nlme - gls	MASS - glm.nb	log nlme - gls	MASS - glm.nb

Supplementary Table 9 | Model Summaries – Gestational age-related variables

	Fannyhessea vaginae	***Gardnerella leopoldii***	***Gardnerella vaginalis***	***Lactobacillus crispatus***	***Lactobacillus gasseri***	***Lactobacillus iners***	***Lactobacillus jensenii***
**Gestational age (weeks) at sample, n=324**							
Estimate	-1·042E-01	1·018E-01	-7·344E-03	5·023E-01	-2·758E-01	-2·320E-01	-3·100E-01
p.values	7·057E-01	3·634E-01	9·691E-01	5·208E-03	3·774E-02	2·144E-01	8·019E-02
FDR corrected p.values (q.values)	8·234E-01	5·088E-01	9·691E-01	**3·646E-02**	1·321E-01	3·752E-01	1·871E-01
Model	MASS - glm.nb	nlme - gls	nlme - gls	nlme - gls	MASS - glm.nb	nlme - gls	MASS - glm.nb
**Gestational age (weeks) at sample, stratified by nulliparity (Nulliparous (n=188))**							
Estimate	-1·684E-01	-3·720E-01	-2·182E-01	6·742E-01	2·562E-01	-1·424E-01	-4·384E-01
p.values	6·852E-01	2·377E-02	3·846E-01	6·426E-03	1·431E-01	4·711E-01	5·357E-02
FDR corrected p.values (q.values)	6·852E-01	8·320E-02	5·384E-01	**4·498E-02**	2·505E-01	5·496E-01	1·250E-01
Model	MASS - glm.nb	MASS - glm.nb	nlme - gls	nlme - gls	MASS - glm.nb	MASS - glm.nb	MASS - glm.nb
**Gestational age (weeks) at sample, stratified by nulliparity (Multiparous (n=136))**							
Estimate	1·185E-01	2·371E-01	3·337E-01	3·318E-02	-4·414E-01	2·079E-01	-1·765E-01
p.values	5·386E-01	1·182E-01	2·589E-01	8·562E-01	8·728E-02	4·558E-01	5·340E-01
FDR corrected p.values (q.values)	6·283E-01	4·138E-01	6·040E-01	8·562E-01	4·138E-01	6·283E-01	6·283E-01

Model	nlme - gls	nlme - gls	nlme - gls	MASS - glm.nb	nlme - gls	nlme - gls	MASS - glm.nb
**Gestational age (weeks) at sample among nulliparous (n=188), stratified by previous pregnancies (Primigravida (n=139))**							
Estimate	-1·762E-01	-4·046E-03	-2·266E-01	8·369E-01	1·226E+00	-7·149E-01	-7·249E-01
p.values	7·315E-01	6·773E-02	4·586E-01	5·065E-03	4·441E-10	1·730E-02	6·739E-03
FDR corrected p.values (q.values)	7·315E-01	9·482E-02	5·351E-01	**1·572E-02**	**3·109E-09**	**3·027E-02**	**1·572E-02**
Model	MASS - glm.nb	log nlme - gls	nlme - gls	nlme - gls	MASS - glm.nb	nlme - gls	MASS - glm.nb
**Gestational age (weeks) at sample among nulliparous (n=188), stratified by previous pregnancies (Nulliparous multigravida (n=49))**							
Estimate	-7·777E-01	1·281E-01	-3·954E-01	3·496E-01	-1·442E+00	3·407E-02	7·013E-01
p.values	2·577E-01	8·935E-01	3·990E-01	4·084E-01	6·422E-06	9·355E-01	7·830E-02
FDR corrected p.values (q.values)	5·717E-01	9·355E-01	5·717E-01	5·717E-01	**4·496E-05**	9·355E-01	2·741E-01
Model	MASS - glm.nb	MASS - glm.nb	MASS - glm.nb	nlme - gls	MASS - glm.nb	MASS - glm.nb	MASS - glm.nb
**Term (Gestational age <41 weeks): Nulliparous (Reference) vs. Multiparous**							
Mean (Multiparous)	1·082E-02	6·639E-03	1·301E-01	2·482E-01	6·622E-02	3·007E-01	8·896E-02
Mean (Nulliparous)	5·308E-03	1·218E-02	9·896E-02	4·880E-01	1·154E-02	2·504E-01	4·996E-02
FoldChange	2·039E+00	-1·834E+00	1·315E+00	-1·966E+00	5·740E+00	1·201E+00	1·780E+00
Estimate	7·128E-01	-5·986E-01	8·490E-02	-1·412E+00	1·744E+00	6·611E-01	5·769E-01
Estimate FoldChange	2·040E+00	5·496E-01	1·089E+00	2·436E-01	5·719E+00	1·937E+00	1·780E+00
p.values	3·641E-01	1·320E-01	8·896E-01	1·104E-02	1·641E-05	2·522E-01	2·880E-01
FDR corrected p.values (q.values)	4·248E-01	3·080E-01	8·896E-01	**3·864E-02**	**1·149E-04**	4·033E-01	4·033E-01
Model	MASS - glm.nb	MASS - glm.nb	log nlme - gls	log nlme - gls	MASS - glm.nb	log nlme - gls	MASS - glm.nb
**Late term (Gestational age ≥41 weeks): Nulliparous (Reference) vs. Multiparous**							
Mean (Multiparous)	1·018E-02		1·465E-01	2·151E-01	5·635E-02	3·737E-01	3·941E-02
Mean (Nulliparous)	4·816E-03		5·508E-02	6·541E-01	1·298E-02	1·930E-01	1·867E-02
FoldChange	2·114E+00		2·660E+00	-3·041E+00	4·342E+00	1·936E+00	2·111E+00
Estimate	9·289E-01		1·284E+00	-4·390E-01	1·464E+00	1·350E+00	1·986E-01
Estimate FoldChange	2·532E+00		3·610E+00	3·289E-01	4·322E+00	3·859E+00	1·220E+00
p.values	1·272E-02		2·544E-02	7·227E-11	3·015E-04	3·123E-02	6·758E-01
FDR corrected p.values (q.values)	**2·544E-02**		**3·748E-02**	**4·336E-10**	**9·045E-04**	**3·748E-02**	6·758E-01
Model	log nlme - gls		log nlme - gls	nlme - gls	MASS - glm.nb	log nlme - gls	log nlme - gls

Supplementary Table 10 | Model Summaries – Gestational age-related variables adjusted for age, BMI, and smoking

	Fannyhessea vaginae	*Gardnerella leopoldii*	*Gardnerella vaginalis*	*Lactobacillus crispatus*	*Lactobacillus gasseri*	*Lactobacillus iners*	*Lactobacillus jensenii*
**Gestational age (weeks) at sample, stratified by nulliparity (Nulliparous (n=188))**							
Estimate	-7·042E-01	-3·853E-02	-2·917E-01	6·581E-01	2·490E-01	-1·445E-01	-3·196E-01
p.values	0·000E+00	8·130E-01	2·491E-01	9·550E-03	1·630E-01	4·743E-01	1·599E-01
FDR corrected p.values (q.values)	**0·000E+00**	8·130E-01	3·487E-01	**3·343E-02**	2·853E-01	5·533E-01	2·853E-01
Model	glm	nlme - gls	nlme - gls	nlme - gls	MASS - glm.nb	MASS - glm.nb	MASS - glm.nb
**Gestational age (weeks) at sample, stratified by nulliparity (Multiparous (n=136))**							
Estimate	1·102E-01	8·437E-04	3·317E-01	4·488E-02	-4·934E-01	NA	-1·027E-01
p.values	7·592E-01	6·101E-01	2·486E-01	8·082E-01	5·875E-02	NA	6·722E-01
FDR corrected p.values (q.values)	8·082E-01	8·082E-01	7·457E-01	8·082E-01	3·525E-01	NA	8·082E-01
Model	MASS - glm.nb	log nlme - gls	nlme - gls	MASS - glm.nb	nlme - gls	None	nlme - gls
**Gestational age (weeks) at sample among nulliparous (n=188), stratified by previous pregnancies (Primigravida (n=139))**							
Estimate	-7·227E-01	9·355E-02	-2·699E-01	7·839E-01	1·208E+00	-1·695E-01	-7·605E-01
p.values	0·000E+00	6·134E-01	3·826E-01	1·029E-02	1·268E-09	4·566E-01	4·579E-03
FDR corrected p.values (q.values)	**0·000E+00**	6·134E-01	5·327E-01	**1·800E-02**	**4·439E-09**	5·327E-01	**1·068E-02**
Model	glm	nlme - gls	nlme - gls	nlme - gls	MASS - glm.nb	MASS - glm.nb	MASS - glm.nb
**Gestational age (weeks) at sample among nulliparous (n=188), stratified by previous pregnancies (Nulliparous multigravida (n=49))**							
Estimate	-1·543E+00	-7·682E-01	-6·204E-01	4·504E-01	-2·259E-01	-5·742E-02	8·407E-01
p.values	3·090E-02	4·233E-01	1·820E-01	2·825E-01	1·717E-172	8·943E-01	3·082E-02
FDR corrected p.values (q.values)	7·209E-02	4·939E-01	3·186E-01	3·955E-01	**1·202E-171**	8·943E-01	7·209E-02
Model	MASS - glm.nb	MASS - glm.nb	MASS - glm.nb	nlme - gls	MASS - glm.nb	MASS - glm.nb	MASS - glm.nb

Supplementary Table 11 | Model Summaries – Demographic background variables

	Fannyhessea vaginae	*Gardnerella leopoldii*	*Gardnerella vaginalis*	*Lactobacillus crispatus*	*Lactobacillus gasseri*	*Lactobacillus iners*	*Lactobacillus jensenii*
**Higher education: No (Reference) vs. University or polytechnic**							
Mean (University or polytechnic)	5·586E-03	3·238E-03	8·573E-02	4·790E-01	3·945E-02	2·212E-01	5·789E-02
Mean (No)	9·568E-03	1·272E-02	1·175E-01	3·766E-01	2·735E-02	3·232E-01	2·432E-02
FoldChange	-1·713E+00	-3·927E+00	-1·371E+00	1·272E+00	1·443E+00	-1·461E+00	2·380E+00
Estimate	-5·382E-01	-1·351E+00	-1·320E+00	8·255E-01	3·656E-01	-1·687E+00	8·671E-01
Estimate FoldChange	5·838E-01	2·590E-01	2·671E-01	2·283E+00	1·441E+00	1·850E-01	2·380E+00
p.values	4·117E-01	1·345E-06	2·299E-03	5·668E-02	2·454E-01	1·127E-04	3·426E-02
FDR corrected p.values (q.values)	4·117E-01	**9·415E-06**	**5·364E-03**	7·936E-02	2·863E-01	**3·946E-04**	5·996E-02
Model	MASS - glm.nb	MASS - glm.nb	log nlme - gls	log nlme - gls	MASS - glm.nb	log nlme - gls	MASS - glm.nb
**Current smoking: No (Reference) vs. Yes**							
Mean (Yes)	1·645E-02	1·633E-02	8·808E-02	2·307E-01	8·820E-04	4·546E-01	7·105E-03
Mean (No)	6·625E-03	6·349E-03	1·008E-01	4·418E-01	3·419E-02	2·610E-01	4·671E-02
FoldChange	2·482E+00	2·572E+00	-1·144E+00	-1·915E+00	-3·876E+01	1·742E+00	-6·575E+00
Estimate	9·092E-01	1·177E+00	1·333E+00	-8·788E-01	-3·562E+00	3·023E+00	-1·883E+00
Estimate FoldChange	2·482E+00	3·244E+00	3·794E+00	4·153E-01	2·837E-02	2·055E+01	1·522E-01
p.values	5·991E-01	2·147E-01	2·273E-01	4·232E-01	1·997E-05	1·112E-03	9·135E-02
FDR corrected p.values (q.values)	5·991E-01	3·183E-01	3·183E-01	4·937E-01	**1·398E-04**	**3·892E-03**	2·131E-01
Model	MASS - glm.nb	log nlme - gls	log nlme - gls	log nlme - gls	MASS - glm.nb	log nlme - gls	MASS - glm.nb
**History of smoking: No (Reference) vs. Yes**							
Mean (Yes)	7·269E-03	7·965E-03	8·263E-02	4·607E-01	1·692E-02	3·028E-01	3·233E-02
Mean (No)	6·508E-03	5·781E-03	1·101E-01	4·266E-01	4·809E-02	2·251E-01	5·560E-02
FoldChange	1·117E+00	1·378E+00	-1·332E+00	1·080E+00	-2·842E+00	1·345E+00	-1·720E+00
Estimate	1·106E-01	3·189E-01	6·640E-02	1·523E-01	-1·041E+00	1·473E+00	-5·421E-01
Estimate FoldChange	1·117E+00	1·376E+00	1·069E+00	1·164E+00	3·531E-01	4·360E+00	5·815E-01
p.values	8·605E-01	2·475E-01	8·718E-01	7·139E-01	5·096E-04	4·706E-04	1·734E-01
FDR corrected p.values (q.values)	8·718E-01	4·330E-01	8·718E-01	8·718E-01	**1·784E-03**	**1·784E-03**	4·045E-01
Model	MASS - glm.nb	MASS - glm.nb	log nlme - gls	log nlme - gls	MASS - glm.nb	log nlme - gls	MASS - glm.nb
**BMI, kg/m2**							
Estimate	2·194E-02	4·443E-02	1·309E-01	1·253E-02	1·613E-02	3·555E-03	-3·551E-02
p.values	7·320E-01	1·213E-01	2·421E-03	7·579E-01	6·001E-01	9·332E-01	3·880E-01

FDR corrected p.values (q.values)	8·842E-01	4·245E-01	**1·695E-02**	8·842E-01	8·842E-01	9·332E-01	8·842E-01
Model	MASS - glm.nb	nlme - gls	nlme - gls	nlme - gls	MASS - glm.nb	nlme - gls	MASS - glm.nb
**Sex partners during lifetime: 3 or less (Reference) vs over 3**							
Mean (over 3)	6·558E-03	5·500E-03	7·422E-02	4·606E-01	2·772E-02	2·920E-01	4·382E-02
Mean (3 or less)	9·196E-03	8·791E-03	1·401E-01	4·441E-01	4·413E-02	1·502E-01	5·272E-02
FoldChange	1·402E+00	1·598E+00	1·888E+00	-1·037E+00	1·592E+00	-1·944E+00	1·203E+00
Estimate	-3·337E-01	-4·631E-01	-2·499E-01	2·200E-01	-4·638E-01	1·681E+00	-1·848E-01
Estimate FoldChange	7·163E-01	6·293E-01	7·789E-01	1·246E+00	6·289E-01	5·369E+00	8·313E-01
p.values	2·589E-01	1·276E-01	6·040E-01	6·301E-01	1·643E-01	1·683E-04	6·743E-01
FDR corrected p.values (q.values)	4·530E-01	3·833E-01	6·743E-01	6·743E-01	3·833E-01	**1·178E-03**	6·743E-01
Model	MASS - glm.nb	MASS - glm.nb	log nlme - gls	log nlme - gls	MASS - glm.nb	log nlme - gls	MASS - glm.nb
**Infertility treatments before current pregnancy: No (Reference) vs. Yes**							
Mean (Yes)	2·975E-04	6·837E-03	9·557E-02	4·711E-01	5·398E-02	1·725E-01	7·726E-02
Mean (No)	7·411E-03	6·644E-03	1·007E-01	4·326E-01	3·164E-02	2·738E-01	4·318E-02
FoldChange	-2·491E+01	1·029E+00	-1·054E+00	1·089E+00	1·706E+00	-1·587E+00	1·789E+00
Estimate	-3·071E+00	2·577E-02	-1·140E+00	2·265E-01	9·226E-01	-1·267E+00	5·819E-01
Estimate FoldChange	4·637E-02	1·026E+00	3·198E-01	1·254E+00	2·516E+00	2·817E-01	1·789E+00
p.values	1·119E-09	9·607E-01	1·586E-01	7·737E-01	2·212E-01	1·032E-01	4·485E-01
FDR corrected p.values (q.values)	**7·833E-09**	9·607E-01	3·700E-01	9·026E-01	3·872E-01	3·610E-01	6·278E-01
Model	MASS - glm.nb	MASS - glm.nb	log nlme - gls	log nlme - gls	log nlme - gls	log nlme - gls	MASS - glm.nb
**Infertility treatments ever: No (Reference) vs. Yes**							
Mean (Yes)	1·146E-03	5·460E-03	1·054E-01	4·518E-01	4·507E-02	1·888E-01	8·111E-02
Mean (No)	8·111E-03	7·244E-03	1·020E-01	4·315E-01	3·529E-02	2·660E-01	4·049E-02
FoldChange	-7·080E+00	-1·327E+00	1·033E+00	1·047E+00	1·277E+00	-1·409E+00	2·003E+00
Estimate	-1·921E+00	-2·830E-01	-1·007E+00	5·138E-02	6·297E-01	-1·236E+00	6·946E-01
Estimate FoldChange	1·464E-01	7·535E-01	3·652E-01	1·053E+00	1·877E+00	2·907E-01	2·003E+00
p.values	1·371E-06	4·986E-01	1·279E-01	9·358E-01	2·621E-01	5·984E-02	2·525E-01
FDR corrected p.values (q.values)	**9·597E-06**	5·816E-01	2·984E-01	9·358E-01	3·670E-01	2·095E-01	3·670E-01
Model	MASS - glm.nb	MASS - glm.nb	log nlme - gls	log nlme - gls	log nlme - gls	log nlme - gls	MASS - glm.nb
**Intercourse <48 hours prior to sample: No (Reference) vs. Yes**							
Mean (Yes)	1·699E-03	1·278E-02	8·205E-02	5·412E-01	1·049E-02	2·077E-01	6·610E-02
Mean (No)	7·927E-03	5·627E-03	1·012E-01	4·312E-01	3·807E-02	2·610E-01	4·223E-02
FoldChange	-4·666E+00	2·272E+00	-1·234E+00	1·255E+00	-3·631E+00	-1·256E+00	1·565E+00
Estimate	-1·541E+00	8·121E-01	4·204E-02	6·066E-01	-1·285E+00	-1·565E-01	4·480E-01

Estimate FoldChange	2·142E-01	2·253E+00	1·043E+00	1·834E+00	2·767E-01	8·551E-01	1·565E+00
p.values	7·258E-02	3·161E-02	9·423E-01	2·945E-01	1·904E-03	7·909E-01	4·206E-01
FDR corrected p.values (q.values)	1·694E-01	1·106E-01	9·423E-01	5·153E-01	**1·333E-02**	9·227E-01	5·889E-01
Model	MASS - glm.nb	MASS - glm.nb	log nlme - gls	log nlme - gls	MASS - glm.nb	log nlme - gls	MASS - glm.nb
**Use of probiotics: No (Reference) vs. Yes**							
Mean (Yes)	6·717E-03	4·165E-03	9·379E-02	4·604E-01	3·651E-02	2·491E-01	4·272E-02
Mean (No)	7·531E-03	1·142E-02	1·135E-01	4·236E-01	3·359E-02	2·449E-01	5·176E-02
FoldChange	-1·121E+00	-2·743E+00	-1·211E+00	1·087E+00	1·087E+00	1·017E+00	-1·212E+00
Estimate	-1·114E-01	-9·965E-01	-6·211E-01	1·347E-01	8·347E-02	1·057E-01	-1·920E-01
Estimate FoldChange	8·945E-01	3·692E-01	5·374E-01	1·144E+00	1·087E+00	1·111E+00	8·253E-01
p.values	6·835E-01	3·679E-04	1·541E-01	7·500E-01	7·882E-01	8·114E-01	6·393E-01
FDR corrected p.values (q.values)	8·114E-01	**2·575E-03**	5·393E-01	8·114E-01	8·114E-01	8·114E-01	8·114E-01
Model	MASS - glm.nb	MASS - glm.nb	log nlme - gls	log nlme - gls	MASS - glm.nb	log nlme - gls	MASS - glm.nb
